# Multicohort Validation of Gut Microbiome Signatures for Cholangiocarcinoma Diagnosis and Functional Characterization of *Bifidobacterium Pseudocatenulatum*

**DOI:** 10.1002/advs.202517658

**Published:** 2026-03-12

**Authors:** Benchen Rao, Jianwen Jiang, Ruiqing Zhang, Dongya Zhang, Chenchen Zhang, Ang Li, Haifeng Lu, Hua Zhang, Lin Zhou, Wenzhi Guo, Peihao Wen, Jianfeng Xue, Jie Pan, Tuerganaili Aji, Zhou Lan, Xianzhi Jiang, Shusen Zheng, Zujiang Yu, Zhigang Ren

**Affiliations:** ^1^ Department of Infectious Diseases State Key Laboratory of Antiviral Drugs Pingyuan Laboratory the First Affiliated Hospital of Zhengzhou University Zhengzhou China; ^2^ Gene Hospital of Henan Province/Precision Medicine Center the First Affiliated Hospital of Zhengzhou University Zhengzhou China; ^3^ Department of Hepatobiliary and Pancreatic Surgery The First Affiliated Hospital Zhejiang University School of Medicine Hangzhou China; ^4^ Department of Hepatobiliary and Echinococcosis Surgery Digestive and Vascular Surgery Center First Affiliated Hospital of Xinjiang Medical University Urumqi China; ^5^ Microbiome Research Center Moon (Guangzhou) Biotech Ltd Guangzhou China; ^6^ State Key Laboratory for Diagnosis and Treatment of Infectious Diseases National Clinical Research Center for Infectious Diseases National Medical Center for Infectious Diseases Collaborative Innovation Center for Diagnosis and Treatment of Infectious Diseases The First Affiliated Hospital Zhejiang University School of Medicine Hangzhou, Zhejiang province China; ^7^ Department of Hepatobiliary and Pancreatic Surgery The First Affiliated Hospital of Zhengzhou University Zhengzhou China

**Keywords:** cholangiocarcinoma, diagnostic model, gut microbiome, liver cancer, metagenome

## Abstract

Growing evidence suggests a role for the gut microbiome in progression of cholangiocarcinoma (CCA), however, its diagnostic and therapeutic potential remains incompletely characterized. Here, metagenomic sequencing was performed on fecal samples (*n* = 785) from individuals across East, Central, and Northwestern China. Gut microbial dysbiosis in CCA was characterized by depletion of short‐chain fatty acids‐producing species and enrichment of potential pathobionts (*Klebsiella aerogenes*, *Clostridium symbiosum*). Diagnostic models built using species‐level markers demonstrated superior performance, compared to pathway‐based models, achieving area under the curve (AUC) values of 98.63% and 99.42% in the discovery cohort, with robust cross‐regional validation (AUC = 80.89% and 80.43%). The model effectively distinguished CCA from hepatocellular carcinoma (AUC = 97.86%) and liver fibrosis (AUC = 98.73%) and nonalcoholic fatty liver disease (mean AUC = 96.86%). Analysis of public datasets encompassing 6847 samples across 31 studies and 11 disease states revealed moderate disease specificity influenced by biomarker overlap across conditions. Mechanistically, depleted *Bifidobacterium pseudocatenulatum* suppressed CCA progression, associated with inhibition of the PI3K‐AKT‐mTOR pathway. Collectively, this study supports the potential of fecal metagenomic signatures as a complementary noninvasive aid for CCA detection, and provides functional evidence for a candidate protective microbe.

## Graphical abstract

This study analyzes gut bacteria in cholangiocarcinoma patients, revealing distinct microbial signatures that enable accurate disease detection. Species‐based diagnostic models achieved over 98% accuracy in identifying cholangiocarcinoma and distinguished it from other liver diseases. The research demonstrates that specific beneficial bacteria suppress tumor progression through key cellular pathways, establishing gut microbiome analysis as a promising noninvasive diagnostic and therapeutic approach.

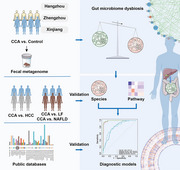

## 1 Introduction

Cholangiocarcinoma (CCA) is the second most common primary liver cancer, accounting for up to 20% [1]. And the prevalence and mortality of cholangiocarcinoma have increased in recent years [2]. CCA is sporadic, and the risk factors show regional differences. In Southeast Asia, common risk factors for CCA are liver flukes (*Opisthorcis viverrini* and *Clonorchis sinensis*), chronic typhoid carriage, and chronic intraductal gallstones, as well as Choledochal cysts and Caroli's disease (intrahepatic biliary cysts) [3]. However, in Western countries, the most important risk factor is primary sclerosing cholangitis [4]. There are other risk factors, including radiation [5], hepatitis B virus or hepatitis C virus infection, and cirrhosis [6, 7]. However, no obvious predisposing factors are found in most patients diagnosed with CCA. Due to the lack of effective diagnostic tools and specific clinical symptoms for early stage CCA patients, most CCA patients are diagnosed in the advanced stage. Resection surgery, considered the only possible curative treatment, is available for only 20%–30% of CCA patients. The postoperative recurrence rate is high (49%–64%), and the current prognosis of CCA patients is still poor [1, 8, 9]. Therefore, identifying new potential therapeutic targets, establishing noninvasive diagnostic tools are of great significance for improving prognosis of CCA patients.

Gut microbiome has been shown to be involved in the occurrence and progression of various hepatobiliary diseases. The “biliary‐enteric circulation” and “liver‐gut microbiome” axis are closely linked to hepatobiliary and intestinal functions [10]. The liver transports bile salts and antibacterial molecules (IgA and angiopoietin) to the gut through the biliary tract to control bacterial overgrowth and maintain gut microbial balance. Then, bile salts, as important signaling molecules regulate processes such as bile acid synthesis, glucose metabolism, lipid metabolism, and dietary energy utilization in the liver through receptors such as FXR and GPBAR1. In addition, intestinal products such as host or microbial metabolites and microbial‐associated molecular patterns can be translocated to the liver via the portal vein and affect liver function. Moreover, hepatic metabolites transported to the intestine via the capillary system can have an impact on the intestinal barrier [11]. Therefore, gut microbial dysbiosis can promote the occurrence and development of liver diseases. On the other hand, studies have shown that gut microbial dysbiosis is a common feature of different independent risk factors for CCA (primary sclerosing cholangitis, liver fluke infection, gallstones, etc.) [12, 13, 14]. Therefore, revealing the role of gut microbiome in the occurrence and development of CCA has important clinical significance for the precise prevention and treatment of CCA.

Cholangiocarcinoma is difficult to diagnose because of its anatomic location, growth patterns, and lack of definite diagnostic criteria [3]. Although new diagnostic biomarkers [15] are frequently discovered and reported, there are many limitations. Liver biopsy is the gold standard for the diagnosis of CCA, however, it's an invasive procedure and sometimes this procedure cannot be performed. So, it's urgent to find novel and noninvasive diagnostic biomarkers for CCA. Gut microbiome is used as a diagnostic biomarker in many diseases, including colorectal cancer, type 2 diabetes, and autoimmune hepatitis. In our previous studies, we constructed a diagnostic model based on 30 gut microbial markers for hepatocellular carcinoma (HCC) and achieved cross‐regional validation in different regions of China [16]. Several studies have explored gut microbiota alterations in CCA patients using 16S rRNA sequencing [17, 18]. While these pioneering investigations provided preliminary evidence for microbiome‐based diagnosis, they were limited by small sample sizes (84–140 subjects), single‐center designs, genus‐level taxonomic resolution, and lack of independent cross‐regional validation. Establishing a robust diagnostic model based on large‐scale metagenomic data from multiple regions with rigorous external validation remains an urgent unmet need. Beyond diagnostic model construction, systematic assessment of disease specificity across multiple conditions and functional validation of key microbial species are essential steps toward clinical translation that have not been addressed in CCA.

Here, we characterized gut microbial metagenomic signatures in 785 participants across three distinct geographical regions in China (East, Central, and Northwest). Using shotgun metagenomic sequencing, we achieved species‐level resolution and comprehensive functional pathway profiling. We constructed and validated noninvasive diagnostic models in multiple independent cross‐regional cohorts, evaluated their capacity to differentiate CCA from HCC and liver fibrosis, and assessed disease specificity using publicly available datasets encompassing 6847 samples across 31 studies and 11 disease states. Finally, we conducted functional validation and mechanism exploration for key species in CCA.

## 2 Results

### 2.1 Cohort and Clinical Characteristics

The samples of this study came from three different regions: Hangzhou (discovery cohort), Zhengzhou (independent validation cohort), and Xinjiang (independent validation cohort). We randomly divided samples from Hangzhou into a training phase (97 CCA and 105 Control) and a testing phase (42 CCA and 45 Control) at a ratio of 70% and 30%. In the training phase, we determined the overview of gut microbial profiles in patients with CCA. Then, the association between differential microbial taxa, MetaCyc‐pathways, and clinical phenotypes is identified. Key species and pathway biomarkers were figured out and used to establish diagnostic models for CCA, respectively. Moreover, the testing phase and two independent cross‐region validation cohort were evaluated the diagnostic efficiency, respectively. Furthermore, we further evaluated the model's potential to differentiate between HCC and CCA, as well as between benign hepatobiliary diseases and CCA. Finally, metagenomic data from 6847 samples in publicly available datasets were used to further verify the model.

We calculated the clinical characteristics of each cohort. In each cohort, the CCA and Control groups were matched for sex and age (Table 1). In the training phase, there were no significant differences in height, weight, body mass index (BMI), total bilirubin (TB), direct bilirubin (DB), and globulin (GLO) between the two groups. The CCA group had worse liver function, with lower albumin (ALB), indirect bilirubin (IB), total protein (TP), and platelets (PLT), and higher alanine aminotransferase (ALT), aspartate aminotransferase (AST), Child‐Turcotte‐Pugh (CTP) score, and glutamyl transpeptidase (GGT). Moreover, Carbohydrate antigen 19‐9 (CA19‐9) was significantly enriched in the CCA group. We further analyzed the comorbidities of the participants, the results showed that hypertension (23.7%), chronic hepatitis B (CHB, 18.6%), and type 2 diabetes (T2DM, 11.3%) were the most common comorbidities in patients with CCA. Similar statistical results can be obtained from other cohorts. The detailed clinical data were provided in Table S2.

**TABLE 1:** Cohort and clinical characteristics.

	Discovery cohort from Hangzhou						Independent validation cohort from Zhengzhou			Independent validation cohort from Xinjiang		
	Training phase			Testing phase								
Characteristic	CCA *N* = 97	Control *N* = 105	*p*‐value	CCA *N* = 42	Control *N* = 45	*p*‐value	CCA *N* = 105	Control *N* = 112	*p*‐value	CCA *N* = 45	Control *N* = 50	*p*‐value
Gender			0.933			0.195			0.761			0.948
Male	56 (58%)	60 (57%)		29 (69%)	25 (56%)		64 (61%)	66 (59%)		24 (53%)	27 (54%)	
Female	41 (42%)	45 (43%)		13 (31%)	20 (44%)		41 (39%)	46 (41%)		21 (47%)	23 (46%)	
Age (years)	58 (52, 63)	56 (45, 72)	0.931	58 (54, 67)	55 (32, 67)	0.124	61 (54, 67)	60 (55, 66)	0.394	60 (54, 69)	56 (53, 67)	0.235
Height (cm)	165 (160, 171)	166 (157, 170)	0.495	168 (164, 174)	165 (157, 175)	0.370	165 (160, 170)	166 (159, 172)	0.810	165 (160, 170)	165 (159, 173)	0.641
Weight (kg)	63 (59, 70)	66 (57, 76)	0.318	70 (62, 75)	69 (59, 76)	0.862	61 (55, 70)	72 (58, 77)	0.010	62 (55, 69)	69 (60, 75)	0.047
BMI (kg/m^2)	23.7 (22.2, 25.2)	23.9 (21.6, 26. 8)	0.258	24.5 (23.1, 25.2)	24.4 (22.2, 27.6)	0.668	23.2 (20.5, 25.0)	25.6 (23.5, 27.4)	0.001	23.1 (21.0, 25.2)	24.1 (23.1, 26.3)	0.025
Tumor diameter (cm)	6 (3, 11)	N/A		9 (4, 25)	N/A		3.0 (2.2, 4.2)	N/A		3.1 (2.0, 5.0)	N/A	
Location of lesion												
iCCA	60 (62%)	N/A		18 (43%)	N/A		42 (40%)	N/A		19 (42%)	N/A	
pCCA	26 (27%)	N/A		17 (40%)	N/A		46 (44%)	N/A		13 (29%)	N/A	
dCCA	11 (11%)	N/A		7 (17%)	N/A		17 (16%)	N/A		13 (29%)	N/A	
Differentiation												
1	5 (6%)	N/A		4 (15%)	N/A		8 (8.9%)	N/A		6 (17%)	N/A	
2	26 (35%)	N/A		11 (42%)	N/A		41 (46%)	N/A		11 (31%)	N/A	
3	27 (36%)	N/A		9 (35%)	N/A		29 (32%)	N/A		10 (29%)	N/A	
4	17 (23%)	N/A		2 (7.7%)	N/A		12 (13%)	N/A		8 (23%)	N/A	
ALT (U/L)	28 (16, 47)	20 (14, 29)	0.001	40 (27, 105)	19 (14, 29)	<0.001	37 (19, 101)	16 (11, 21)	<0.001	66 (30, 126)	18 (15, 25)	<0.001
AST (U/L)	28 (24, 44)	22 (19, 26)	<0.001	34 (25, 71)	19 (17, 24)	<0.001	44 (22, 77)	20 (16, 23)	<0.001	59 (29, 95)	21 (19, 23)	<0.001
GGT (U/L)	72 (42, 169)	22 (16, 35)	<0.001	100 (55, 310)	20 (14, 36)	<0.001	121 (31, 521)	18 (12, 24)	<0.001	265 (124, 496)	22 (15, 28)	<0.001
TP (g/L)	67 (64, 71)	76 (73, 78)	<0.001	65 (63, 73)	77 (73, 79)	<0.001	65 (60, 70)	73 (71, 76)	<0.001	68 (63, 72)	72 (65, 76)	0.017
ALB (g/L)	39 (36, 42)	48.6 (46.9, 50.3)	<0.001	39 (34, 42)	48.5 (46.9, 50.4)	<0.001	38 (33, 41)	47 (45, 49)	<0.001	35.6 (31.5, 38.3)	46.1 (43.1, 47.7)	<0.001
GLO (g/L)	28.1 (24.6, 32.1)	27.7 (24.1, 30.3)	0.212	27.6 (24.3, 30.5)	28.9 (26.1, 30.8)	0.718	27.1 (24.0, 31.1)	26.4 (23.6, 28.7)	0.190	31 (27, 37)	25 (22, 29)	<0.001
TB (µmol/L)	11 (8, 23)	12 (9, 15)	0.745	17 (9, 100)	12 (7, 16)	0.004	25 (10, 136)	10 (8, 13)	<0.001	32 (11, 160)	8 (6, 12)	<0.001
DB (µmol/L)	5 (4, 16)	5 (4, 6)	0.031	6 (4, 85)	4 (3, 6)	<0.001	13 (5, 104)	4 (3, 6)	<0.001	20 (5, 98)	3 (2, 4)	<0.001
IB (µmol/L)	6 (4, 10)	7.2 (5.1, 9.6)	0.059	10 (5, 15)	7 (4, 10)	0.159	9 (5, 20)	6 (5, 8)	0.019	11 (6, 39)	5 (4, 7)	<0.001
CTP score	6.00 (5.00, 8.00)	5.00 (5.00, 5.00)	<0.001	7.00 (6.00, 8.00)	5.00 (5.00, 5.00)	<0.001	6.00 (5.00, 7.00)	5.00 (5.00, 5.00)	<0.001	7.00 (6.00, 8.00)	5.00 (5.00, 5.00)	<0.001
PLT (10^9/L)	187 (150, 226)	236 (209, 279)	<0.001	187 (159, 233)	234 (203, 290)	0.001	190 (122, 230)	230 (194, 250)	<0.001	268 (179, 298)	217 (184, 247)	0.041
CA199 (U/mL)	128 (17, 512)	8 (7, 11)	<0.001	324 (24, 1,156)	7 (5, 11)	<0.001	95 (23, 535)	8 (6, 11)	<0.001	149 (43, 1,654)	8 (6, 11)	<0.001
Comorbidities												
Hypertension	23 (23.7%)	N/A		12 (28.6%)	N/A		27 (25.7%)	N/A		15 (33.3%)	N/A	
T2DM	11 (11.3%)	N/A		2 (12.0%)	N/A		12 (11.4%)	N/A		5 (11.1%)	N/A	
CAD	5 (5.2%)	N/A		2 (4.8%)	N/A		11 (10.5%)	N/A		3 (6.7%)	N/A	
LC	10 (10.3%)	N/A		3 (7.1%)	N/A		11 (10.5%)	N/A		3 (6.7%)	N/A	
CHB	18 (18.6%)	N/A		6 (14.3%)	N/A		11 (10.5%)	N/A		6 (13.3%)	N/A	
Cerebrovascular disease	5 (5.2%)	N/A		1 (2.4%)	N/A		5 (4.7%)	N/A		2 (4.4%)	N/A	

Data are median (IQR). *p* values calculated by Pearson's Chi‐square test, Wilcoxon rank sum test or Fisher's exact test. *p*‐value < 0.05 considered statistically significant.

Abbreviations: ALB, albumin; ALT, Alanine aminotransferase; AST, Aspartate aminotransferase; BMI, Body mass index; CA19‐9, Carbohydrate antigen 19‐9; CAD, Coronary heart disease; CCA, cholangiocarcinoma; CHB, chronic hepatitis B; CTP, Child‐Turcotte‐Pugh; DB, direct bilirubin; dCCA, distal cholangiocarcinoma; GGT, Glutamyl transpeptidase; GLO, globulin; IB, indirect bilirubin; iCCA, intrahepatic cholangiocarcinoma; LC, liver cirrhosis. N/A, not available; pCCA, perihilar cholangiocarcinoma; PLT, platelets; T2DM, type 2 diabetes; TB, total bilirubin; TP, total protein.

### 2.2 Gut Microbial Dysbiosis Was Detectable in CCA

To investigate the gut microbiome in patients with CCA, we analyzed the data from metagenomic shotgun sequencing. The Shannon index, Simpson index and Invsimpson index was used to demonstrate the α diversity between two groups (Figure S1A–C, Figure S2A–C). The results based on the species level showed that the Shannon index of the CCA group were lower than those of the Control group (*p*‐value < 0.01, adjusted by Microbiome Multivariable Association with Linear Models, MaAsLin2) (Figure S1A), which was contrary to the results of previous study with small sample size [17]. Then, the β diversity was found significantly increased in the CCA group compared to the Control group according to Bray distance (*p*‐value < 0.01) (Figure S1D,E). Principal coordinate analysis (PCoA) and adjusted principal coordinate analysis (aPCoA) showed that the samples of CCA group and Control group were obviously separated, showing that the overall gut microbial composition was different between the two groups (Figures S1F–G and S2D,E).

**FIGURE 1:**
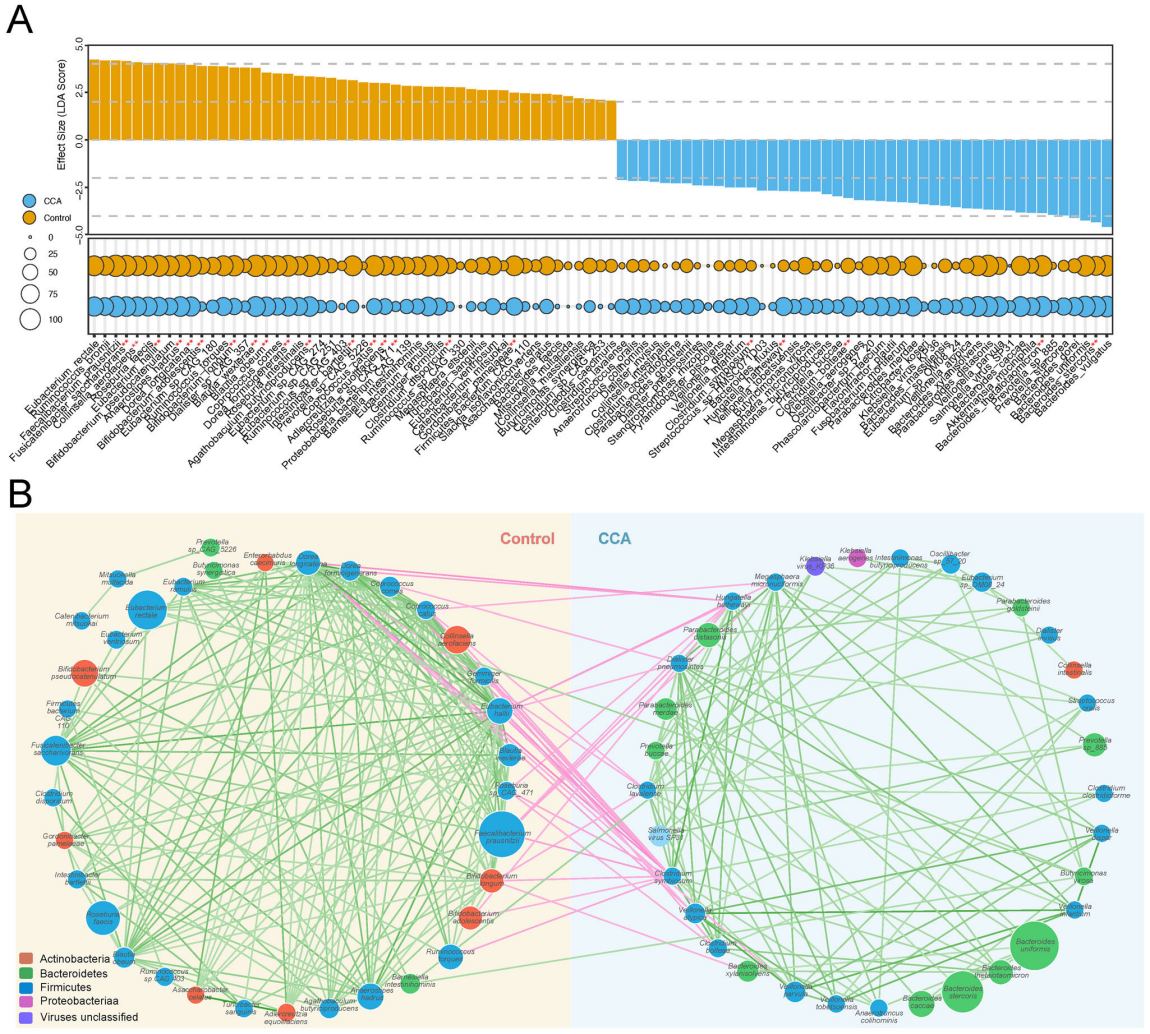
**Significantly different gut bacteria between cholangiocarcinoma (CCA) and Control subjects**. (A) Detection frequency and linear discriminant analysis (LDA) score of the significantly different gut bacteria (species level) in CCA and Control groups using Wilcoxon rank sum test, linear discriminant analysis effect size (LEfSe) and Microbiome Multivariable Association with Linear Models (MaAsLin2) were shown. Represents the species incorporated in the following diagnostic model. (B) Co‐occurrence network was deduced from significantly different species. Nodes depict species with their taxonomic information displayed in the center and colored according to phylum. The size of the nodes indicated relative abundance of the species. Connecting lines represent Spearman correlation coefficient values >=0.4 (green) or <= −0.4 (purple).

Subsequently, the composition and comparison of the gut microbial community were analyzed. We demonstrate the dominant microbiome by calculating the average composition. At the genus level, the abundance of *Bacteroides*, *Prevotella*, *Roseburia*, *Eubacterium*, *Faecalibacterium*, *Alistipes*, and *Escherichia* accounted for more than 50% of all genera in both groups (Figure S2F). Similar results can be approached at the species level (Figure S1H), indicating that CCA disease status has little impact on the dominant species of gut microbial community. Additionally, we identified differential microbiota at different levels by Wilcoxon rank sum test, LEfSe and MaAsLin2. The results illustrated, at the species level, 46 species including *Parabacteroides distasonis*, *Bacteroides stercoris*, *Prevotella stercorea*, *Bacteroides thetaiotaomicron*, *Akkermansia muciniphila* were enriched in the CCA group. On the contrary, 49 species including *Bifidobacterium adolescentis*, *Bifidobacterium pseudocatenulatum*, *Bifidobacterium longum* were enriched in the Control group (Figure 1A, Table S3). At the genus level, we found that *Actinomyces*, *Lactobacillus* were depleted in the CCA group (Figure S3A, Table S4), which was contrary to previous study with small sample size. Jia et al. proposed that *Actinomyces* and *Lactobacillus* were significantly enriched in iCCA [17], while our study included CCA of all locations. Moreover, we conducted co‐abundance network analysis by Spearman correlations between species. The correlation network analysis revealed distinct microbial signatures between CCA and Control groups. Species enriched in the Control group predominantly included SCFAs‐producing taxa such as *Faecalibacterium prausnitzii*, *Roseburia faecis*, and *Eubacterium hallii*, which exhibited dense positive correlations within their ecological cluster. Conversely, case‐enriched species featured potential pathobionts including *Clostridium bolteae*, *Veillonella dispar*, and *Klebsiella aerogenes*, demonstrating strong intra‐group positive connectivity (Figure 1B). Notably, anti‐correlative relationships were observed between the two clusters, particularly between *Bifidobacterium adolescentis* (Control‐enriched) and *Clostridium symbiosum* (CCA‐enriched). Similar results can be approached at the genus level (Figure S3B).

### 2.3 Gut Microbial MetaCyc‐pathway was Detectable in CCA and was Associated with Clinical Phenotypes

Studies have reported that gut microbial metabolites were closely related to the occurrence and progression of liver cancer, in which alterations in bacterial gene function play an important role [19]. Therefore, we further analyzed the characteristics of gut microbial MetaCyc‐pathway in patients with CCA based on the MetaCyc database. Significantly decreased α diversity and β diversity were found in the CCA group versus the Control group (*p*‐value < 0.01) (Figure S4A–E). The results of PCoA showed that the pathway of the two groups were significantly separated in the PCoA1 axis (85.02%, *p*‐value < 0.01), and then there was no significant difference in the PCoA2 axis (5.6%, *p*‐value > 0.05) (Figure S4F). Then, we used Wilcoxon rank sum test and MaAsLin2 to identify the differential pathways according to the *z*‐score of relative abundance. The results illustrated that the urea cycle and glycolysis (from fructose 6‐phosphate) were significantly upregulated in the CCA group, and the pentose phosphate pathway was significantly downregulated compared with the Control group (Figure S4G, Table S5). Previous studies have shown that elevated glycolysis were closely related to colon cancer [20], and *Fusobacterium nucleatum* in the intestine promoted tumorigenesis by increasing the sugar metabolism of colon cancer cells [21]. Notably, the biosynthetic pathways of amino acids such as l‐aspartic acid, l‐asparagine, and l‐citrulline were elevated. On the contrary, the biosynthesis of aromatic amino acids, l‐arginine, and l‐glutamine were depleted in the CCA group. In addition, the biosynthesis of nucleotides such as adenosine nucleotides, guanosine nucleotides, adenosine deoxyribonucleotides, and guanosine deoxyribonucleotides was significantly increased. Moreover, the biosynthesis of palmitates was significantly downregulated in the CCA group. In summary, our results suggest that patients with CCA have unique microbial metabolic processes.

**FIGURE 2:**
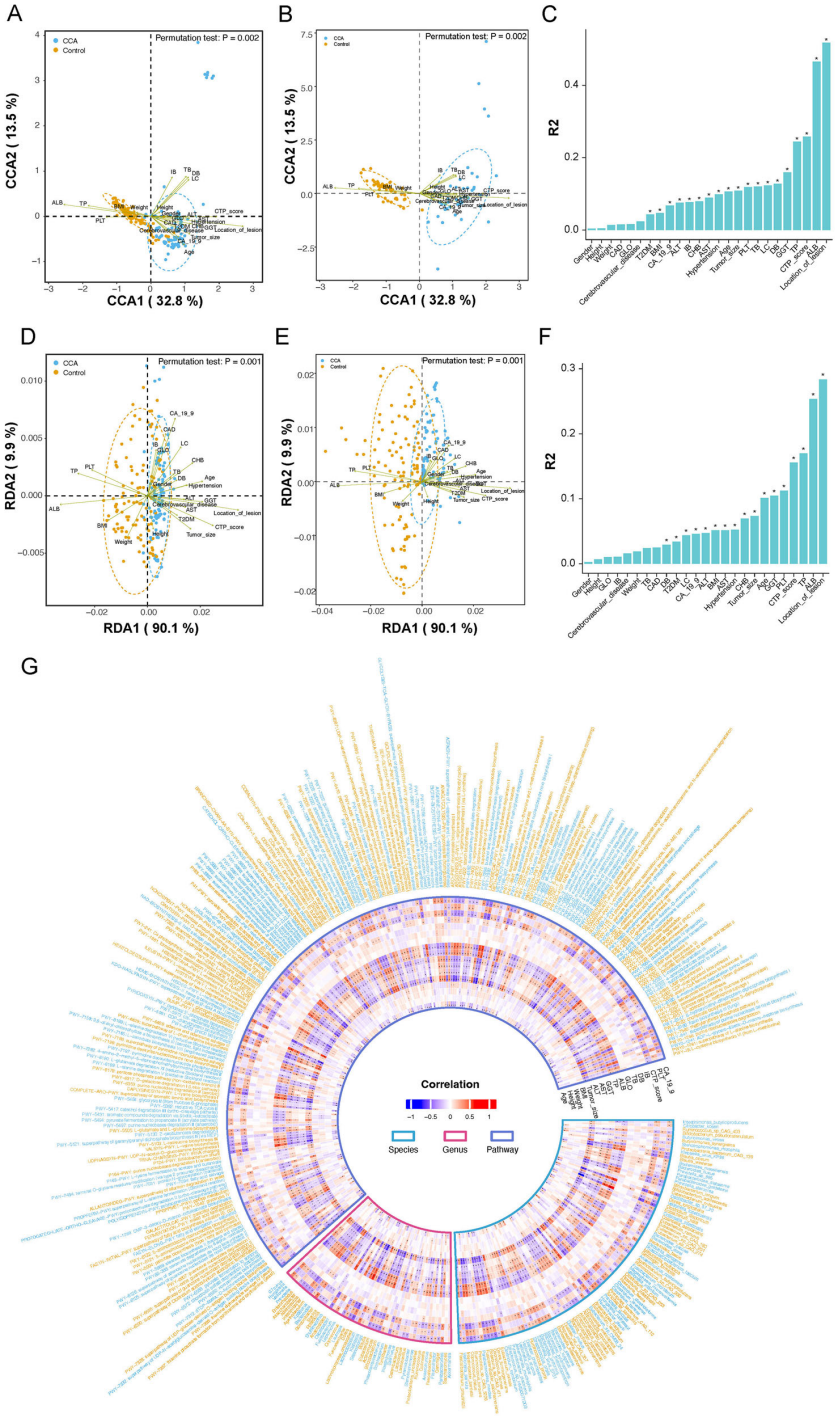
**Correlation analysis between gut microbiome and clinical phenotypes**. (A, B) Canonical correspondence analysis with permutation test showed the clinical factors associated with the distribution of all samples based on the different species. (C) Contribution of different clinical factors to canonical correspondence analysis for all differential taxa (^*^ *p*‐value < 0.05). (D,E) Redundancy analysis (RDA) with permutation test displayed the clinical factors associated with the distribution of all samples based on the different MetaCyc‐pathway. (F) Contribution of different clinical factors to RDA for all differential MetaCyc‐pathway (^*^ *p*‐value < 0.05). (G) Spearman's rank correlation analysis between gut microbiome (species, genus and MetaCyc‐pathways level) and clinical factors (^+^ *p*‐value < 0.05, ^*^ *p*‐value < 0.01).

To explore the correlation between gut microbiome and clinical phenotypes in patients with CCA, we investigated the characteristics of clinical phenotypes in the train set. Canonical correspondence analysis revealed that samples separated primarily along the vector for CCA status, with the first two axes explaining 32.8% and 13.5% of the total variance, respectively (permutation test *p* = 0.002, Figure 2A). Redundancy analysis corroborated this finding, showing that CCA status explained 90.1% of the constrained variance in the first axis (RDA1), with clear separation between CCA and control samples (permutation test *p* = 0.001, Figure 2B). These results confirm that the presence of CCA is the dominant factor shaping gut microbiome composition, rather than demographic variables or comorbidities. In addition to CCA status, the four phenotypes that have the greatest impact on species were location of lesion, ALB, CTP score, and TP (Figure 2C, Table S6). Notably, age, sex, BMI, and presence of common comorbidities (hypertension, diabetes, CAD, cerebrovascular disease) showed minimal effects, supporting the disease‐specificity of the microbiome signature. Analyses at the functional pathway level (MetaCyc pathways) yielded highly concordant results. Canonical correspondence analysis and RDA both showed significant separation by CCA status (Figure 2D,E), with location of lesion, ALB, TP, and CTP score again emerging as the top phenotypic determinants (Figure 2F, Table S7). The concordance between species‐level and pathway‐level results indicate coordinated taxonomic and functional responses to clinical phenotypes. A Spearman's rank correlation analysis between gut microbiome (species, genus and MetaCyc pathways) and clinical phenotypes was conducted (Figure 2G, Tables S8 and S9). Importantly, tumor size showed significant positive correlations with several species previously implicated in carcinogenesis. *Flavonifractor plautii* and *Clostridium bolteae* were the species most strongly associated with larger tumors. These bile‐tolerant bacteria are involved in secondary bile acid metabolism and production of proinflammatory metabolites. Conversely, tumor size showed negative correlations with beneficial commensals including *Faecalibacterium prausnitzii* and *Akkermansia muciniphila*. In summary, these findings suggest that specific gut microbiome features may be linked to CCA tumor progression, potentially through production of protumorigenic metabolites and loss of tumor‐suppressive metabolites.

### 2.4 Noninvasive Diagnosis for CCA Based on the Gut Microbiome

**FIGURE 3:**
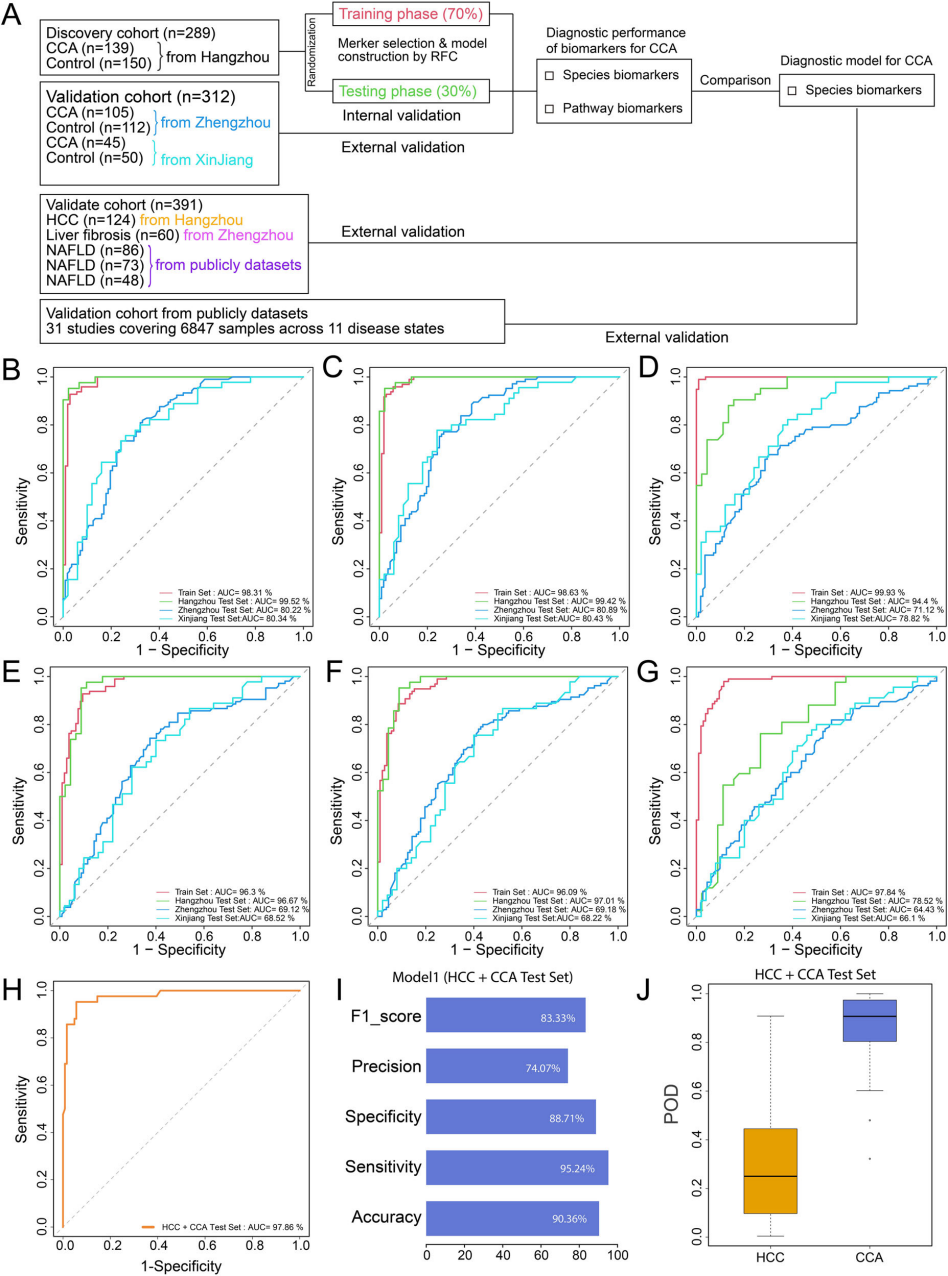
**Gut microbial unconstrained diagnostic model (model‐1) for cholangiocarcinoma (CCA)**. (A) Flow diagram of establishing and validating of the diagnostic model. Samples from Hangzhou were used as the discovery cohort and randomly divided into a training phase and a testing phase at a ratio of 70% and 30%. Random forest model was conducted to establish diagnostic models based on training phase by species markers and MetaCyc‐pathway markers, respectively. And internal validation was performed on testing phase. Then, two independent cross‐regional validation cohort from Zhengzhou and Xinjiang demonstrated the diagnostic potential of the model. The models were further evaluated to distinguish between CCA from hepatocellular carcinoma (HCC) and benign hepatobiliary diseases. Moreover, a validation cohort from publicly datasets covering 6847 samples across 11 disease states was evaluated the disease specificity of the best diagnostic model. The optimal species‐sets (including both CCA‐enriched and Control‐enriched species) were used to construct unconstrained models by (B) random forest (RF) algorithms, (C) weighted random forest (WRF) algorithms and (D) balanced random forest (BRF) algorithms. The optimal pathway‐sets (including both CCA‐enriched and Control‐enriched pathways) were used to construct unconstrained models by (E) RF algorithms, (F) WRF algorithms and (G) BRF algorithms. (H) ROC curve, (I) prediction performance and (J) probability of diseases (POD) index of “WRF‐species‐model‐1” to distinguish between CCA and HCC.

To illustrate the noninvasive diagnostic value of gut microbiome for CCA, we constructed random forest classifiers that could specifically identify CCA. We randomly divided samples from Hangzhou, China into a training phase (97 CCA and 105 Control) and a testing phase (42 CCA and 45 Control) at a ratio of 70% and 30% (Figure 3A). To ensure the selection of the most appropriate algorithm for CCA diagnosis, we conducted a comprehensive comparison of 17 machine learning algorithms spanning multiple methodological categories, including Random Forest (RF), Gradient Boosting Machine (GBM), Extreme Gradient Boosting (XGBoost), Recursive Partitioning and Regression Trees (rpart), Ridge Regression, Lasso Regression, Elastic Net (Enet) with varying alpha parameters (*α* = 0.1, 0.2, 0.3, 0.4, 0.5, 0.6, 0.7, 0.8, 0.9), Generalized Linear Model (glm), Support Vector Machine (SVM) (Figure S5). The results show that the RF algorithm yields the highest average AUC on the test set. Thus, we selected RF as the foundation algorithm for subsequent model development. Twenty‐two species were chosen as the optimal species set to establish the “RF‐species‐model‐1” (model‐1: unconstrained model) (Table S10). The discovery cohort obtained AUC values of 98.31% and 99.52% in the training phase and testing phase, respectively, indicating high diagnostic efficiency for CCA (Figure 3B, Tables S11 and S12). A previous study indicated that microbiome‐based diagnostic models cannot ignore regional differences [22]. Thus, two independent cross‐regional validation sets were recruited from Zhengzhou (105 CCA and 112 Control) and Xinjiang (45 CCA and 50 Control) to demonstrate the diagnostic potential of the model. Importantly, Zhengzhou test set achieved an AUC value of 80.22% (Table S13). Strikingly, Xinjiang test set showed an AUC value of 80.34% (Table S14).

In this study, the number of samples in the Control group was greater than that in the case group, which may lead to potential problems of unbalanced cohorts and affecting the performance of the machine learning model. Therefore, we constructed models using the weighted random forest (WRF) algorithm and the balance random forest (BRF) algorithm, respectively. The results showed that the prediction performance of the “WRF‐species‐model‐1” (Figure 3C, Table S15) was better than that of the “RF‐species‐model‐1” and “BRF‐species‐model‐1” (Figure 3D, Table S16). The AUCs of the training set and the three test sets were 98.63%, 99.42%, 80.89%, and 80.43%, respectively by the “WRF‐species‐model‐1”. And the “WRF‐species‐model‐1” was established by 22 species including *Clostridium symbiosum*, *Dorea formicigenerans*, *Eubacterium hallii*, and *Hungatella hathewayi*, the mean decrease in accuracy demonstrated that *Eubacterium hallii* may be the most predominant marker in this diagnostic model (Figure S6A–F).

Then, we further explored the potential of pathways as diagnostic markers for CCA. Eighteen pathway markers were selected to construct the “RF‐pathway‐model‐1” (Table S17). In the discovery cohort, high diagnostic efficiencies were still obtained (96.3% and 96.67%), but only 69.12% and 68.52% were received in two independent cross‐regional validation sets (Figure 3E, Table S18). Moreover, the prediction performance of the “WRF‐pathway‐model‐1” (Figure 3F, Table S19) was similar to that of the “RF‐pathway‐model‐1”, while the results of the “BRF‐pathway‐model‐1” were worse (Figure 3G, Table S20).

Previous studies have shown that some species that are reduced in disease groups relative to Control, such as alterations in some probiotics, are associated with intestinal inflammation and are not disease‐specific [23, 24]. In this study, we found that the abundance of *Bifidobacterium adolescentis*, *Bifidobacterium pseudocatenulatum*, and *Bifidobacterium longum* were significantly reduced in the CCA group compared with the Control group. Therefore, we retrained a classifier and set constraints to select only microbial features that were positively correlated (enriched) in the CCA group to generate enrichment‐constrained model‐2 (constrained to positive features). For model‐2s (enrichment‐constrained model using only CCA‐enriched features), feature selection yielded an optimal subset of eight species (Table S21) for “RF‐species‐model‐2”. On total, we used species markers and pathway markers to construct six model‐2s based on RF, WRF, and BRF algorithms (Figure S7A–F, Tables S22–S27). The results showed that “RF‐species‐model‐2” (Figure S7A) was the best species‐based model‐2, and “WRF‐pathway‐model‐2” (Figure S7E) were the best pathway‐based model‐2, respectively. Moreover, “RF‐species‐model‐2” established by eight species has better diagnostic performance, the most important characteristics were *Hungatella hathewayi*, *Prevotella stercorea*, *Clostridium symbiosum* (Figure S6G–L).

To clarify the general applicability of our model across different subtypes of CCA, we categorized CCA patients according to the anatomical location: iCCA (*n* = 140), pCCA (*n* = 101), and dCCA (*n* = 48). Alpha diversity analysis and PCoA revealed no significant differences in microbial community richness and microbial community structures among the three subtypes (Figure S8A,B). The results showed that “WRF‐species‐model‐1” achieved discriminative efficiencies of 80.7%, 83.2%, and 81.3% across the three subtypes, respectively, demonstrating robust and consistent diagnostic performance (Figure S8C). “WRF‐species‐model‐2” showed relatively lower but still acceptable performance across all three subtypes. In addition, CA19‐9 is an important biomarker for diagnosing CCA. Based on CA19‐9 levels, CCA patients were divided into a CA19‐9 positive group (CA19‐9 ≥ 37, n = 184) and a CA19‐9 negative group (CA19‐9< 37, *n* = 82). Twenty‐three CCA patients with CA19‐9 deficiency were not included in the analysis. The results showed that “WRF‐species‐model‐1” achieved a discrimination efficiency of 81.5% in the CA19‐9 positive group. More importantly, 76.8% of CA19‐9 negative CCA patients could also be identified using the microbial model (Figure S8D). In this study, the CA19‐9 positivity rate was 69.2%, and “WRF‐species‐model‐1” achieved a higher recognition efficiency than CA19‐9, indicating that our model can identify some CCA cases that would be missed by CA19‐9 testing alone. Similarly, “WRF‐species‐model‐2” showed relatively low but still acceptable performance across all three models. These results demonstrate that our microbiome‐based diagnostic models maintain consistent performance across different subtypes of cholangiocarcinoma and show promise as a reliable tool for clinical auxiliary diagnosis.

In summary, “WRF‐species‐model‐1” was the best unconstrained model and “RF‐species‐model‐2” was the best enrichment‐constrained model. And the unconstrained model based on the WRF algorithm and species markers was not only effective in eastern China, but also still applicable in central and northwest China.

### 2.5 Exploring the Potential of Diagnostic Models to Distinguish CCA from HCC

In clinical practice, the differential diagnosis between cholangiocarcinoma and hepatocellular carcinoma is a difficult problem in diagnosis and treatment because they have some similar clinical symptoms and imaging manifestations. So, we tried to use the above gut microbial diagnostic model to distinguish CCA from HCC. Patients with HCC (*n* = 124) were included in the analysis. We defined a positive result of the prediction model as CCA and a negative result as HCC. Based on “WRF‐species‐model‐1”, a diagnostic efficiency of 97.86% was obtained (specificity: 88.71%, sensitivity: 95.24%), and the POD index of the CCA group was significantly higher than that of the HCC group (Figure 3H–J, Table S28). However, only 82.33% of the diagnostic efficiency was obtained by “RF‐species‐model‐2” (Figure S7G–I, Table S29). The F1_score, precision, specificity, sensitivity, and accuracy of model 2 were all lower than those of model 1. The results indicated that the unconstrained model based on the WRF algorithm and species markers can not only efficiently diagnose CCA from Control, but also specifically distinguish CCA from HCC.

### 2.6 Exploring the Potential of Diagnostic Models to Distinguish CCA from Benign Hepatobiliary Diseases

To address concerns about false positives from benign hepatobiliary diseases and to evaluate the clinical applicability of our models in real‐world diagnostic scenarios that differentiate between CCA and benign liver diseases. We constructed four validation cohorts, including patients with liver fibrosis (LF, *n* = 60) from the First Affiliated Hospital of Zhengzhou University, and three geographically distinct nonalcoholic fatty liver disease (NAFLD, also known as Metabolic Dysfunction‐Associated Steatotic Liver Disease in recent years) cohorts retrieved from public databases: PRJNA373901 (USA, *n* = 86), PRJEB14215 (Italy, *n* = 73), and PRJNA420817 (Sweden, *n* = 48). These cohorts represent common benign hepatobiliary diseases that share overlapping clinical features with CCA, including abnormal liver function and some clinical symptoms, making them well‐suited for assessing diagnostic specificity. “WRF‐species‐model‐1” demonstrated robust performance across all benign disease cohorts (Figure S9). When differentiating between CCA and LF, the model achieved an AUC of 98.73%, accuracy of 91.18%, sensitivity of 95.24%, and specificity of 88.33% (Figure S9A, Table S30). In the three NAFLD cohorts, the model consistently maintained high performance: PRJNA373901 (AUC = 95.96%, accuracy = 84.38%, Figure S9B), PRJEB14215 (AUC = 94.93%, accuracy = 90.43%, Figure S9C), and PRJNA420817 (AUC = 99.70%, accuracy = 97.78%, Figure S9D). Notably, the Swedish NAFLD cohort exhibited the highest specificity (100%), indicating no false positives, while maintaining a sensitivity of 95.24%. And “RF‐species‐model‐2” also can distinguished between CCA and benign hepatobiliary diseases (Figure S10). Comparative analysis showed that “WRF‐species‐model‐1” consistently outperformed “RF‐species‐model‐2” in all benign liver disease validation cohorts, with a higher average AUC (Table S31). More importantly, “WRF‐species‐model‐1” had a lower false positive rate (FPR), with a specificity exceeding 87% in three of the four cohorts. The results show that the unconstrained model based on the WRF algorithm and species markers can not only effectively distinguish CCA from the control group, but also specifically distinguish CCA from common benign hepatobiliary diseases, which supports their potential clinical application value in complex diagnostic scenarios.

### 2.7 External Validation of the Diagnostic Model on Other Diseases by Publicly Datasets

**FIGURE 4:**
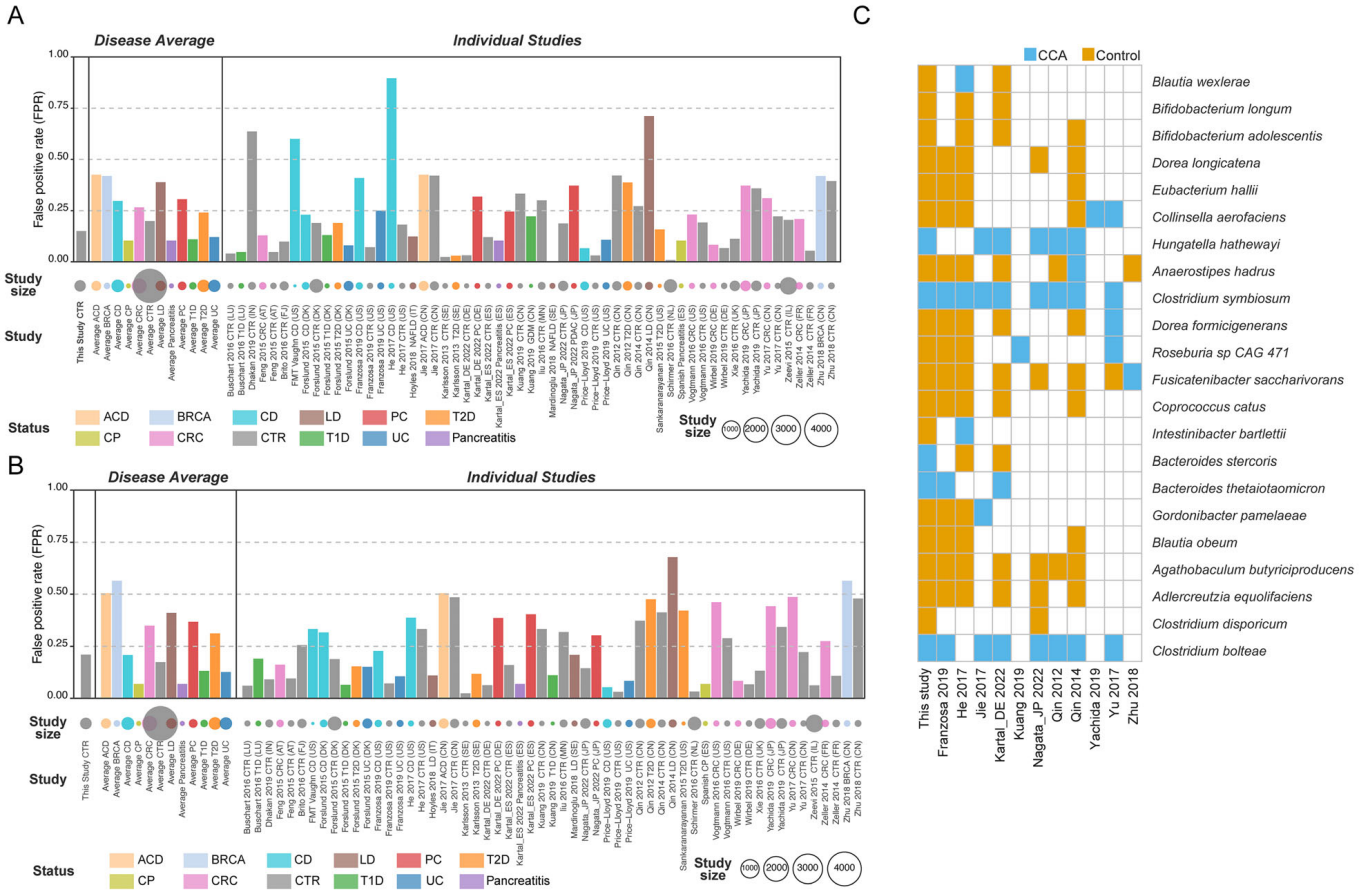
**External validation of the disease specificity of the models**. External validation of the disease specificity of (A) the best unconstrained models and (B) the best enrichment‐constrained models based on cholangiocarcinoma (CCA) enriched features. Bar plot of the false positive rate (FPR) of the predictive model in 31 external validation sets was shown. The number of the subjects in each group is displayed as color coded circles below. (C) The cohorts with FPR greater than 0.3 for the disease group or CTR and containing both disease group and CTR samples were selected to display characteristics of shared markers for “WRF‐species‐model‐1”. WRF‐species‐model‐1, unconstrained model based on significantly different species by weighted random forest algorithms; CRC, colorectal cancer; T2D, type 2 diabetes; T1D, type 1 diabetes; CD, Crohn's disease; UC, ulcerative colitis; LD, liver diseases; ACD, atherosclerotic coronary disease; PC, pancreatic cancer; BRCA, breast cancer; CP, chronic pancreatitis; CTR, healthy controls; CCA, cholangiocarcinoma.

To further evaluate the disease specificity of the diagnostic model based on species markers, we obtained predictions for samples from other available gut metagenomic datasets, as in previous studies [23, 25]. We screened publicly available fecal metagenomic datasets from published case‐control or cohort studies, resulting in data from 31 studies covering 6847 samples in 11 disease states including subjects with colorectal cancer (CRC, *n* = 628), Crohn's disease (CD, *n* = 404), type 2 diabetes (T2D, *n* = 382), ulcerative colitis (UC, *n* = 372), liver diseases (LD, *n* = 239), atherosclerotic coronary disease (ACD, *n* = 214), pancreatic cancer (PC, *n* = 144), type 1 diabetes (T1D, *n* = 91), breast cancer (BRCA, *n* = 62), chronic pancreatitis (CP, *n* = 29), pancreatitis (*n* = 29) as well as healthy controls (CTR, *n* = 4253) (Table S1). The results of “WRF‐species‐model‐1” showed that the FPR of CTR in this study was 0.15 (Figure 4A, Table S32). Overall, the average FPR for CTR in the external validation cohort was 19.89%. Except for BRCA (0.42), CD (0.48), ACD (0.43), LD (0.39) and PC (0.31) the average FPR of other diseases was lower than 0.3. Then, we observed higher FPR in several cohorts of patients. The FPR of CTR in cohorts from India (Dhakan 2019) were higher than 0.5, which may be related to local economic conditions, health conditions, diet, and other factors [26, 27]. Moreover, there were two CD cohorts showed high FPR (He 2017, FMT Vaughn), a LD cohort from China also showed a higher FPR of 0.71 (Qin 2014). Subsequently, we evaluated the performance of “RF‐species‐model‐2” (Figure 4B, Table S33). Consistent with previous studies, the enrichment‐constrained model can reduce the FPR of CTR samples in the external validation cohort [23, 25]. Compared with “WRF‐species‐model‐1”, the FPR was higher in most cohorts in “RF‐species‐model‐2”. To sum up, based on the performance of the two models in the CCA cross‐regional validation cohort, HCC external validation cohort, benign hepatobiliary diseases cohort, and public database external validation cohort, we believe that the unconstrained model based on the WRF algorithm and species markers was the optimal diagnostic model for CCA.

Although the FPR of “WRF‐species‐model‐1” in the external validation cohort of the public database was lower than that of “RF‐species‐model‐2”, the disease specificity requires careful interpretation. The average FPR for healthy controls was 19.89%, which is consistent with performance metrics reported in other microbiome‐based cancer diagnostic models. Importantly, we conducted the most comprehensive disease specificity assessment in the CCA microbiome literature to date, encompassing 6847 samples across 31 studies and 11 disease states. This large scope of validation was deliberately designed to identify the boundaries of our model's clinical applicability. And we tried to analyze the reasons for this phenomenon. The shared markers between diseases may be an important reason. We selected the cohorts with FPR greater than 0.3 for the disease group or CTR and containing both disease group and CTR samples for further analysis. A total of 22 species markers were used to construct model 1. Eight of the 11 cohorts showed obvious characteristics of shared markers (the number of shared markers > 3) with CCA (Figure 4C), this result can largely explain the high FPR in these cohorts. Surprisingly, three of the cohorts had less than three markers in common with CCA, and explaining this was the direction of our next study. Recent articles have reported that the human gut microbiota was mainly composed of two core flora, the basic functional group that was beneficial to health, characterized by fiber fermentation and butyrate production, and the pathogenic functional group that was considered harmful, characterized by virulence/pathogenicity and antibiotic resistance [28]. The characteristics of harmful intestinal flora in patients with diabetes, cardiovascular disease, ankylosing spondylitis, colorectal cancer, inflammatory bowel disease, cirrhosis and schizophrenia were highly similar. These findings can also help explain the high FPR of the model in the external disease validation cohort of this study. On the whole, while demonstrating moderate performance in external validation on public datasets, the current diagnostic model exhibited limitations in disease specificity with elevated FPR, primarily attributed to overlapping biomarkers across certain diseases.

### 2.8 Bifidobacterium Pseudocatenulatum Suppresses CCA Progression Associated with PI3K‐AKT‐mTOR Pathway Inhibition

To address whether the identified microbial alterations have functional consequences beyond correlative associations, we investigated the potential anti‐tumor effects of CCA‐depleted species. Among the Control‐enriched species, multiple Bifidobacterium species ranked highly, including *Bifidobacterium pseudocatenulatum*, *Bifidobacterium adolescentis*, and *Bifidobacterium longum* (Figure 1A), suggesting a protective role of this genus against CCA development. Notably, the abundance of these three species exhibited significant differences across multiple regional cohorts. *B. pseudocatenulatum* was consistently enriched in Control group across all four cohorts, while *B. adolescentis* and *B. longum* showed significant enrichment in three cohorts (excluding Zhengzhou test set) (Figure S11A). To evaluate their anti‐tumor function, we treated human CCA cell line RBE with conditioned media from these three Bifidobacterium species. *B. adolescentis* and *B. longum* conditioned media showed minimal inhibitory effects, however, *B. pseudocatenulatum* conditioned media (*B.p* CM) demonstrated dose‐dependent suppression of RBE cell viability (Figure S11B). This inhibitory effect was confirmed in another human CCA cell line, HCCC‐9810 (Figure S11C). Given that 10% *B.p* CM effectively inhibited cell viability in both CCA cell lines, with stronger suppression in 20% *B.p* CM, we selected *B. pseudocatenulatum* for further study.

**FIGURE 5:**
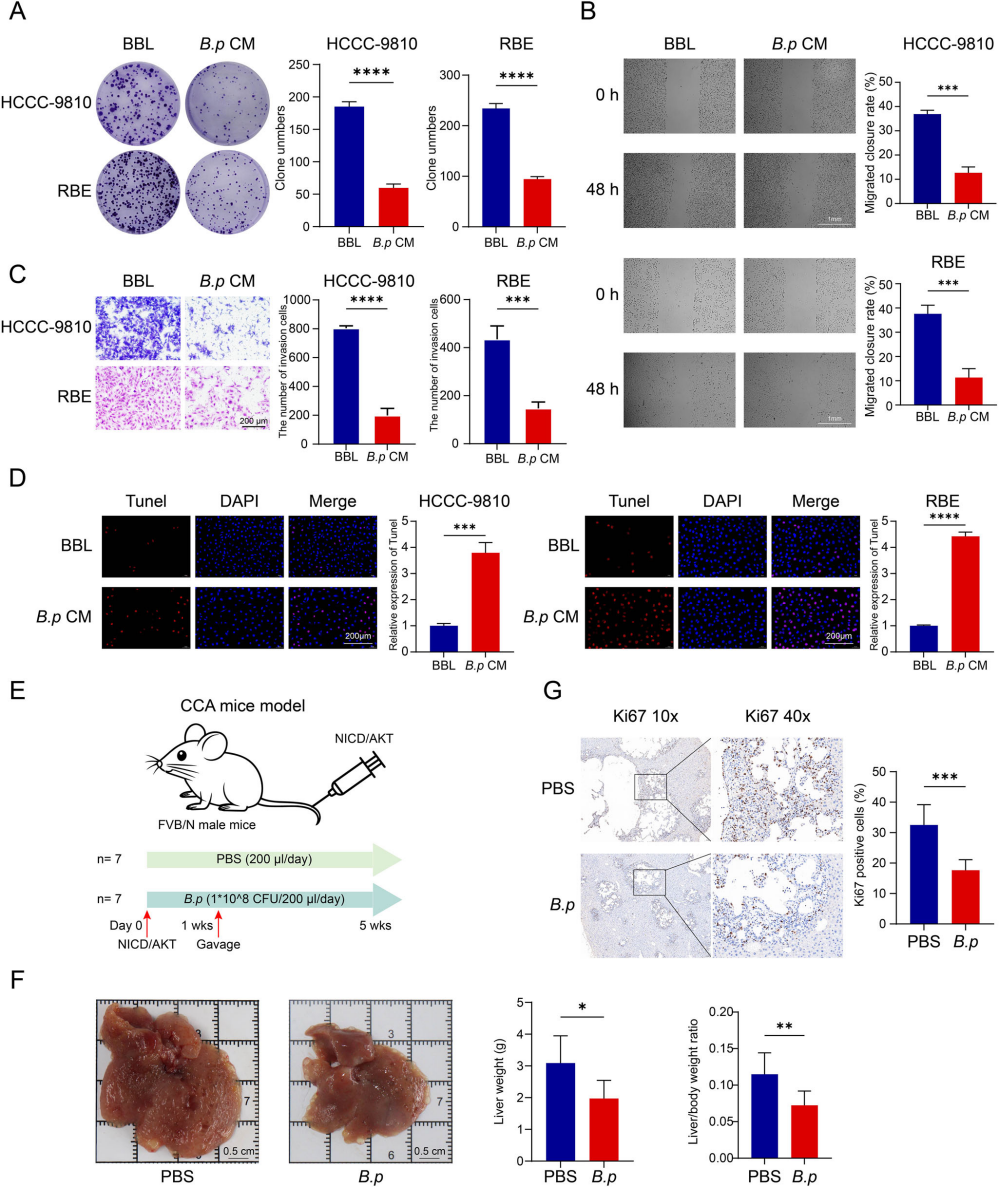
***Bifidobacterium pseudocatenulatum* suppresses cholangiocarcinoma (CCA) malignant phenotypes in vivo and in vitro**. (A) Colony formation assays in HCCC‐9810 and RBE cells treated with Bifidobacterium agar medium (BBL) or *B. pseudocatenulatum* conditioned media (*B.p* CM). (B) Wound healing assays demonstrating migration inhibition. Quantification shows closure rates at 48 h. (C) Transwell invasion assays. (D) TUNEL staining for apoptosis detection. Quantification shows relative expression of TUNEL+ cells. (E) Experimental scheme for CCA mouse model with *B.p* treatment. (F) Representative liver images and quantification of liver weight and liver/body weight ratio. (G) Ki67 immunohistochemistry and quantification.

Then, the effects of *B.p* CM (10%) on CCA cell malignant phenotypes were evaluated using Bifidobacterium agar medium (BBL) as control. Colony formation assays revealed that *B.p* CM significantly reduced clone numbers in both HCCC‐9810 and RBE cells (*p* < 0.0001, Figure 5A). Wound healing assays demonstrated that *B.p* CM significantly suppressed the migratory ability of HCCC‐9810 and RBE cells (*p* < 0.001, Figure 5B). Transwell assays further confirmed that *B.p* CM substantially inhibited the invasive ability of both cell lines (*p* < 0.0001, Figure 5C). Moreover, TUNEL staining revealed that *B.p* CM induced significant apoptosis in HCCC‐9810 (*p* < 0.001) and RBE cells (*p* < 0.0001, Figure 5D).

To validate these findings in vivo, we established a CCA mouse model through hydrodynamic tail vein injection of NICD and AKT oncogenes. Mice were randomly divided into PBS (200 µL/day) group and *B. pseudocatenulatum* (1 × 10^8^ CFU/200 ul/day) group. Treatment began 1 week after modeling and continued for 4 weeks (Figure 5E). Macroscopic examination revealed extensive tumor replacement of liver tissue in PBS group, with large confluent tumor masses. In contrast, *B.p*‐treated mice retained more normal liver architecture with smaller, discrete tumor nodules (Figure 5F). Analysis demonstrated that *B.p* treatment significantly reduced both liver weight (*p* < 0.05) and liver/body weight ratio (*p* < 0.01) compared to PBS group. Ki67 immunohistochemistry showed a significant decrease in proliferative index in *B.p*‐treated tumors (*p* < 0.001, Figure 5G).

Moreover, immunofluorescence staining of colonic tissues revealed that *B.p* treatment significantly enhanced the expression of intestinal barrier proteins, including Claudin 1 (*p* < 0.0001), Occludin (*p* < 0.01), and ZO‐1 (*p* < 0.0001) (Figure S12). Collectively, these data demonstrate that *B. pseudocatenulatum* suppresses CCA progression both in vitro and in vivo while improving intestinal barrier integrity.

**FIGURE 6:**
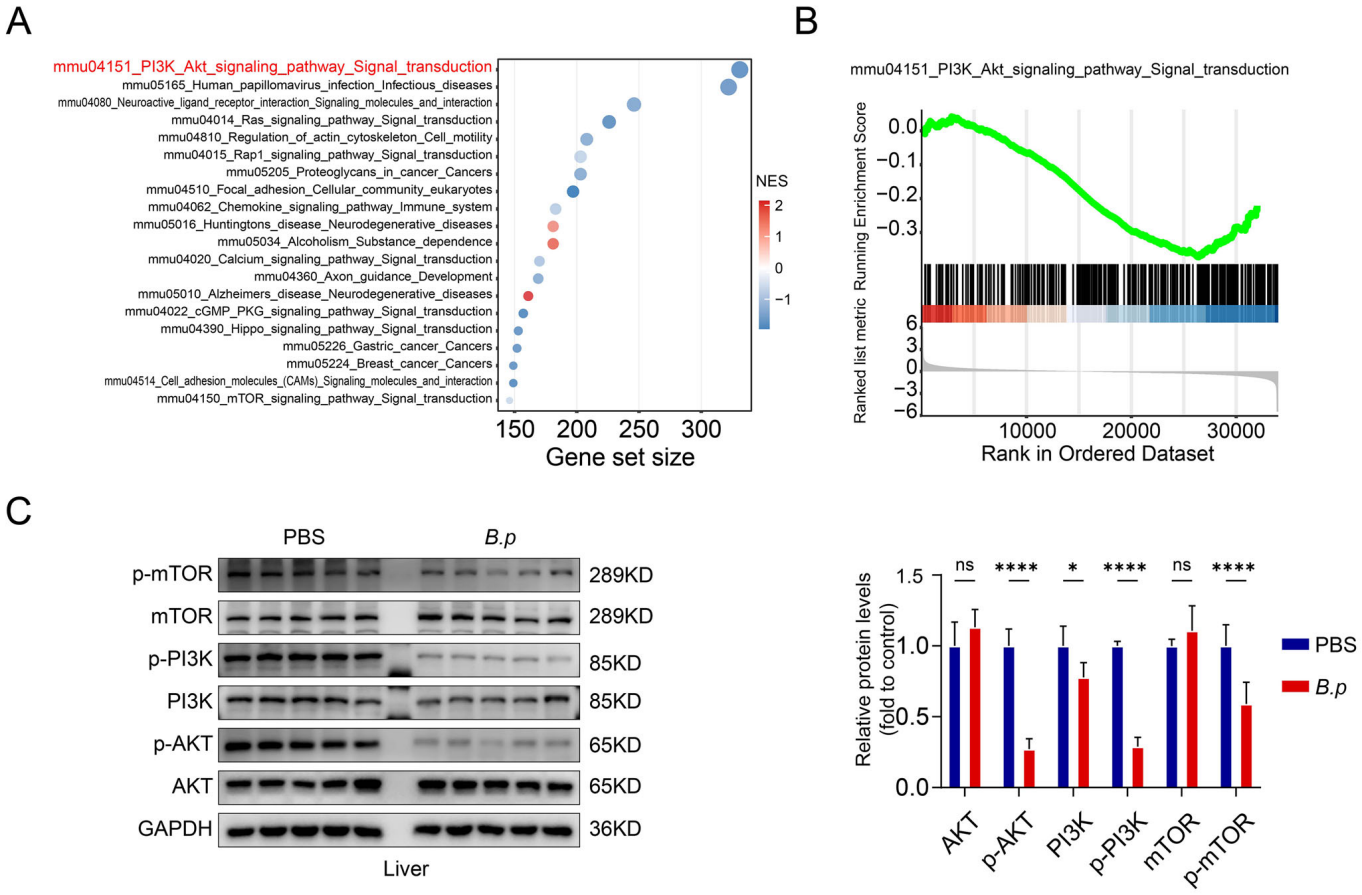
***Bifidobacterium pseudocatenulatum* suppresses cholangiocarcinoma (CCA) progression associated with PI3K‐AKT‐mTOR pathway inhibition**. (A) KEGG pathway enrichment analysis of differentially expressed genes between *Bifidobacterium pseudocatenulatum* (*B.p)* treated and PBS‐treated liver tissues from NICD/AKT‐induced CCA mice. (B) Gene set enrichment analysis (GSEA) plot showing negative enrichment of the PI3K‐AKT signaling pathway in *B.p*‐treated versus PBS‐treated CCA mouse livers. (C) Western blot analysis and quantification of PI3K‐AKT‐mTOR pathway proteins in liver tissues from NICD/AKT‐induced CCA mice treated with PBS or *B.p*. Statistical significance was determined by two‐tailed Student's *t*‐test. ns, not significant; ^*^ *p* < 0.05; ^****^ *p* < 0.0001.

To elucidate the mechanisms underlying *B.p*‐mediated CCA suppression, we performed RNA sequencing on liver tissues from the CCA mouse model treated with either *B.p* or PBS. Gene set enrichment analysis (GSEA) revealed that the PI3K‐AKT signaling pathway (mmu04151) was the most significantly enriched pathway, exhibiting the highest gene count (Figure 6A). The PI3K‐AKT signaling pathway showed significant negative enrichment in the *B.p*‐treated group compared to PBS controls (NES = −0.27, Figure 6B), suggesting that *B.p* suppresses this oncogenic signaling pathway.

To validate these findings, we examined the activation status of this pathway in experimental models. We conducted western blot analysis on liver tissues from NICD/AKT‐induced CCA mice treated with *B.p* or PBS. The result revealed that *B.p* treatment significantly decreased the phosphorylation levels of AKT (*p* < 0.05), PI3K (*p* < 0.0001), and mTOR (*p* < 0.0001) in liver tissues compared to controls (Figure 6C). Interestingly, total protein expression of AKT also showed a significant decrease (*p* < 0.05). In summary, these validations suggest that *Bifidobacterium pseudocatenulatum* suppressed CCA progression, associated with inhibition of the PI3K‐AKT‐mTOR pathway.

## 3 Discussion

Early diagnosis of CCA remains a formidable challenge and an important measure to improve the prognosis of this cancer. The latest CCA diagnosis and management guidelines in 2024 proposed that the current preferred diagnostic methods for CCA were still cytological and histological testing, and there were no other high‐evidence quality biomarkers that have been validated by multiple centers [29]. Small‐scale studies have proposed CCA gut microbial markers for diagnosis by 16S ribosome RNA sequencing with limited applicability.

We acknowledge that the general analytical framework of this study, identifying differentially abundant taxa and constructing machine learning classifiers, follows established approaches in microbiome‐based cancer diagnostics. However, we emphasize that this work makes several substantial contributions beyond simply applying existing methods to a new organ. First, this represents the largest multicenter gut microbiome study specifically designed for CCA diagnosis to date, with 785 participants from three geographically distinct regions, a fivefold to ninefold expansion compared to previous 16S rRNA‐based studies (84–140 subjects) [17, 18]. Second, the employment of shotgun metagenomic sequencing achieved species‐level resolution and comprehensive functional pathway profiling that are inaccessible through 16S approaches, enabling identification of specific pathogenic species that would otherwise be indistinguishable at the genus level. Third, this study conducted rigorous cross‐regional validation of a CCA microbiome classifier, demonstrating consistent performance across populations with different dietary patterns and environmental exposures (AUC 80.89% and 80.43% in Central and Northwest China, respectively). Fourth, we performed comprehensive disease specificity assessment by analyzing 6847 samples across 31 studies and 11 disease states, providing crucial information about cross‐disease marker overlap that was absent from previous CCA studies. Fifth, we addressed clinically important differential diagnosis challenges by demonstrating effective discrimination of CCA from HCC (AUC = 97.86%), liver fibrosis (AUC = 98.73%), and NAFLD, distinctions that have direct clinical relevance given the overlapping presentations of these conditions. Finally, unlike most microbiome‐cancer studies that remain correlative, we provided functional validation demonstrating that *B. pseudocatenulatum* suppresses CCA progression associated with PI3K‐AKT‐mTOR pathway inhibition, establishing causality beyond association.

The concept of the gut microbiome as a noninvasive biomarker tool for disease has been confirmed by outstanding studies. Jun et al. used fecal metagenomic data in patients with CRC to identify CRC‐specific microbial markers and verified them in CRC patients of different races, indicating that alterations in gut microbiota can as a noninvasive diagnostic marker for CRC [30]. Nan et al. analyzed the gut microbial structure of Chinese patients with cirrhosis through metagenomic sequencing, identified 15 markers specific to cirrhosis, and created a highly accurate patient discrimination index [31]. Our previous study characterized the gut microbiome of patients with HCC and constructed a diagnostic model based on 30 microbial markers and validated it in multiple cohorts [16]. In this study, we collected a total of 785 fecal samples from different regions in China completed metagenomic sequencing analysis. Samples from East China were randomly divided into a training set and a test set as a discovery cohort, for the construction and preliminary verification of diagnostic models respectively. Importantly, two independent cross‐regional cohorts from central and northwestern China demonstrated strong diagnostic potential. More importantly, faced with the difficulty of differential diagnosis of CCA and HCC as well as benign hepatobiliary diseases in clinical practice, the model constructed in this study can accurately distinguish CCA from HCC and benign hepatobiliary diseases. Finally, the model's disease specificity was again validated in public datasets covering 6847 samples and 31 studies across 11 disease states. In summary, gut microbiome biomarkers may become noninvasive and efficient diagnostic tools for CCA.

This study identified alterations in gut microbial communities and pathways associated with CCA. There were some interesting features. The most enriched species (with the largest LDA score) in the CCA group was *Bacteroides vulgatus*. Studies have reported that *Bacteroides vulgatus* promotes a proinflammatory environment by producing endotoxins (e.g., LPS) and secondary bile acid accumulation [32, 33]. LPS activated the liver TLR4/NF‐κB signaling pathway, driving the continuous secretion of IL‐6 and TNF‐α, thereby inducing cholangiocyte DNA damage and proliferation [34, 35]. Concurrently, CCA‐enriched *Clostridium botulinum*‐mediated deconjugation of bile acids, promoted the production of tumor‐promoting deoxycholic acid, thereby promoting the generation of reactive oxygen species and the activation of Wnt/β‐catenin [36, 37]. The depletion of butyrate‐producing bacteria (*Faecalibacterium prausnitzii*, *Roseburia intestinalis*) further disrupted immune homeostasis by reducing anti‐inflammatory short‐chain fatty acids, thereby impairing Treg differentiation and enhancing protumorigenic Th17 responses [38, 39]. In addition, Metacyc pathway analysis suggested that CCA‐associated microbiota reshapes host metabolism. Enhanced glycolysis (e.g., glycolysis II, PWY‐5484) and lactate fermentation acidify the microenvironment, promoting angiogenesis and M2 macrophage polarization [40, 41]. In addition, upregulated norspermidine biosynthesis (PWY‐6562) supported polyamine‐driven cell proliferation, while dysregulated arginine metabolism modulates nitric oxide levels, thereby inhibiting apoptosis [42]. These metabolic changes, coupled with the accumulation of secondary bile acids, created a microenvironment that sustains tumor cell survival and immune escape. In summary, we speculate that the gut microbiota may promote the progression of CCA through two interrelated mechanisms: chronic inflammation‐driven carcinogenesis mediated by endotoxins and secondary bile acids, and metabolic reprogramming of the tumor microenvironment to accelerate tumor progression. Based on these mechanisms, the inhibition of LPS/TLR4, supplementation of SCFAs, or modulation of metabolic pathways may provide new therapeutic avenues for CCA. Future studies should verify these interactions in vitro and vivo models and explore their translational potential in the treatment of CCA.

In practical clinical settings, the performance of microbiome‐based diagnostic models should be interpreted in context. In our study, the AUC decreased from 0.98–0.99 in the discovery cohort to ∼0.80 in cross‐regional validation cohorts, a degree of attenuation that is consistent with prior microbiome diagnostic studies and cross‐population validations in colorectal cancer (often showing ∼10%–20% AUC reductions) [43, 44]. Importantly, achieving ∼0.80 AUC in independent cohorts from distinct geographic regions—with differences in diet and environmental exposures—represents a realistic estimate of clinical performance. The model also showed strong discrimination between CCA and several clinically relevant differential diagnoses (AUC 0.9786 vs. HCC, 0.9873 vs. liver fibrosis, mean 0.9686 vs. NAFLD). However, the elevated false‐positive rates observed in some non‐CCA disease cohorts from public databases highlight an important limitation for standalone deployment. The ∼20% average false‐positive rate in non‐CCA samples likely reflects partially shared dysbiosis patterns across inflammatory and metabolic conditions, as summarized in Figure 4C. This observation aligns with the concept that multiple diseases can converge on overlapping, inflammation‐ and metabolism‐associated microbial functional shifts. Accordingly, we position our model as a complementary diagnostic aid rather than a replacement for established clinical workflows. Potential near‐term applications include first‐line screening or triage in higher‐risk groups (e.g., patients with primary sclerosing cholangitis or chronic liver disease, and individuals residing in liver‐fluke–endemic areas), as well as adjunctive support when imaging findings are indeterminate. Looking ahead, integrating microbiome features with host genomics, imaging, and circulating tumor DNA may further improve specificity and clinical utility.

Our functional experiments further suggest that *B. pseudocatenulatum* suppresses CCA progression, associated with reduced PI3K‐AKT‐mTOR signaling. We recognize that probiotic‐associated modulation of PI3K‐AKT‐mTOR has been reported in other cancer contexts [45]. Nevertheless, our data provided disease‐relevant functional support for *B. pseudocatenulatum* in CCA, including validation in a NICD/AKT‐driven model that recapitulates key molecular features of intrahepatic cholangiocarcinoma. The observation that *B. pseudocatenulatum* conditioned medium reduced CCA cell viability, whereas conditioned media from *B. adolescentis* and *B. longum* showed minimal effects (Figure S11B,C), underscores the importance of species‐specific functional evaluation. The specific targeting of the PI3K‐AKT‐mTOR pathway is particularly noteworthy, as aberrant activation of this signaling cascade is prevalent in approximately 40%–50% of intrahepatic CCA and correlates with aggressive tumor behavior and poor patient outcomes [46], supporting the clinical relevance of this pathway. In parallel, the observed improvement in intestinal barrier markers (Claudin‐1, Occludin, and ZO‐1) is consistent with a multifactorial benefit, given proposed links between impaired gut barrier integrity, bacterial translocation, and chronic inflammation in CCA pathogenesis [47]. However, our mechanistic data remained associative rather than definitive, and we did not evaluate CCA‐specific mechanisms such as bile acid transformation or FXR modulation. Future work should test whether *B. pseudocatenulatum* influences hepatobiliary‐relevant pathways (e.g., bile acid–FXR signaling) that could distinguish its effects in CCA from mechanisms described in other cancers. Collectively, our results provide a functional rationale for further evaluating *B. pseudocatenulatum* as a potential adjunctive strategy in CCA.

Although this study has achieved meaningful results, the following limitations should be noted: First, our cohorts were exclusively from Chinese populations. Ethnicity was not recorded; therefore, we cannot evaluate the effect of ethnicity on model performance. We have revised the limitations accordingly and will collect ethnicity and major risk‐factor profiles prospectively in future multi‐country studies. CCA etiology exhibits marked geographical variation, Asian populations are characterized by hepatolithiasis, viral hepatitis, and biliary stones, while Western populations show a higher prevalence of primary sclerosing cholangitis, often associated with inflammatory bowel disease. These etiological differences may influence gut microbiome composition and model performance. Future studies incorporating cohorts from Southeast Asia, Europe, and North America are needed to determine whether our findings represent universal CCA‐associated microbiome signatures or require population‐specific calibration. Additionally, due to the clinical complexity of CCA, detailed risk factor information was not consistently available, limiting our ability to stratify findings by specific etiologies. Second, missing data is a limitation of gut microbiome research. Species annotation based on metagenomic sequencing relies on the integrity of the reference database, and undetected taxa may affect model performance. With the advancement of the Global Microbiome Project, future research is expected to improve detection sensitivity by integrating multiplatform data (such as meta‐transcriptome and metabolome) and further improve species annotation by combining long‐read sequencing and culturing omics technologies. Third, due to incomplete electronic medical record documentation, our dataset lacks smoking and alcohol consumption history, which may have certain implications for the clinical application of the model. Future prospective studies should rigorously collect and handle covariates, employ comprehensive lifestyle questionnaires to quantify these potential influences, and further improve diagnostic accuracy. Fourth, the model of this study still has a high FPR in several external diseases. Although the overlap of markers between diseases can explain this phenomenon, this problem may be overcome in the future through higher resolution sequencing technology. Finally, while our functional validation demonstrated that *B. pseudocatenulatum* suppresses CCA progression associated with PI3K‐AKT‐mTOR pathway inhibition, we did not establish CCA‐specific mechanisms. The demonstration of bile acid transformation, FXR modulation, or other hepatobiliary‐specific pathways would strengthen the mechanistic understanding and distinguish the effects from those reported for other probiotics in different cancer types. Future studies employing metabolomics profiling of bile acids and targeted genetic approaches are warranted to address this limitation.

## 4 Conclusion

In conclusion, this study represents the largest multicenter gut microbiome study specifically designed for CCA diagnosis to date. This study reported the alterations in the gut microbial metagenome of patients with CCA through large cohort data from three different regions in China. This study successfully established and conducted a cross‐regional multicohort cholangiocarcinoma microbial diagnostic model. The model efficiently distinguished CCA from HCC, which is difficult to differentiate in clinical practice, and demonstrated robust performance in discriminating CCA from benign hepatobiliary diseases including liver fibrosis and nonalcoholic fatty liver disease. External validation across international multi‐disease cohorts showed moderate disease specificity, influenced by biomarker overlap across conditions. The model is therefore positioned as a complementary screening and diagnostic aid tool rather than a standalone diagnostic solution, with optimal utility in high‐risk populations and inconclusive clinical scenarios. Beyond diagnostics, functional investigations revealed that *Bifidobacterium pseudocatenulatum* suppresses CCA progression, associated with PI3K‐AKT‐mTOR pathway inhibition and intestinal barrier restoration, providing a preliminary foundation for exploring microbiome‐based therapeutic interventions in CCA.

## 5 Experimental Section

### 5.1 Ethics Statement

This study was conducted based on the prospective specimen collection and retrospective blinded evaluation design principles. This study was approved by the Ethics Committee of the First Affiliated Hospital, School of Medicine, Zhejiang University (East China) (2014–334); the First Affiliated Hospital of Zhengzhou University (Central China) (2017‐XY‐002), and the First Affiliated Hospital of Xinjiang Medical University (Northwestern China) (20170427‐11). Written informed consent was obtained from all participants.

### 5.2 Study Population

Discovery Cohort. Patients with CCA and healthy controls were recruited from the First Affiliated Hospital, School of Medicine, Zhejiang University from October 2015 to May 2016 and December 2024 to February 2025. All enrolled patients were diagnosed with CCA by pathology. Healthy controls were recruited from population who had been confirmed to have no common acute or chronic diseases through physical examinations at the Health Management Center. Patients with CCA from three anatomical parts (intrahepatic cholangiocarcinoma, perihilar cholangiocarcinoma, and distal cholangiocarcinoma) were included in this study after pathological diagnosis. The demographics and clinicopathological data of the participants were collected from hospital electronic medical records and questionnaires. The data on the comorbidities of patients were collected. Finally, 139 patients with CCA and 150 gender‐age‐mated healthy controls were recruited.

Independent Validation Cohort from Zhengzhou. To independently validate the performance of the selected markers and trained models, metagenomics analysis was conducted on an independent cohort from the First Affiliated Hospital of Zhengzhou University. Using the same inclusion and exclusion criteria as the discovery cohort, 105 patients with CCA and 112 gender‐age‐matched healthy controls were enrolled from November 2018 to June 2019 and December 2024 to February 2025.

Independent Validation Cohort from Xinjiang. To further validate the efficiency of diagnostic models, an independent cohort from the First Affiliated Hospital of Xinjiang Medical University was recruited. According to the same inclusion and exclusion criteria, 45 patients with CCA and 50 gender‐age‐matched healthy controls were recruited from March 2019 to August 2019 and December 2024 to February 2025.

Independent Validation Cohort of HCC from Hangzhou. To further explore the potential of diagnostic models to distinguish CCA from HCC, an independent cohort of HCC (*n* = 124) from the First Affiliated Hospital, School of Medicine, Zhejiang University in 2014 were recruited by pathology diagnosis.

Independent Validation Cohort of Benign Hepatobiliary Disease. To evaluate model specificity in distinguishing CCA from benign hepatobiliary diseases, we assembled four independent validation cohorts. Liver fibrosis (LF) samples (*n* = 60) were collected from The First Affiliated Hospital of Zhengzhou University following identical protocols for sample collection, DNA extraction, and metagenomic sequencing as the CCA cohorts. LF diagnosis was confirmed by comprehensive clinical assessment including imaging, laboratory tests, and FibroScan measurements. Three additional NAFLD cohorts were obtained from public repositories: PRJNA373901 (*n* = 86, USA) [48], PRJEB14215 (*n* = 73, Italy) [49], and PRJNA420817 (*n* = 48, Sweden) [50]. Raw sequencing data were processed using our established bioinformatics pipeline to ensure consistency with CCA cohort analyses.

### 5.3 Metagenomic Sequencing

The metagenomic sequencing was conducted on the Illumina NovaSeq 6000 platform. Fecal samples from different regions were sequenced using the same platform. Raw reads were quality trimmed using fastp (v0.19.7) and filtered against the human genome (hg19) using Bowtie2 (v2.3.4) [51]. After quality trimming and filtering, 76.5 million reads per sample on average had remained and used in downstream analyses. Taxonomic profiling was performed with MetaPhlAn3 (v3.0.1) [52], while HUMAnN3 (v3.0.0) was used to estimate MetaCyc‐pathway profiling [52]. The raw sequencing data for all samples were uploaded to the European Nucleotide Archive (ENA) at the European Bioinformatics Institute (EBI) under accession number PRJNA1111706.

### 5.4 CCA Mouse Model

Male FVB/N mice (6 weeks old) were purchased from Charles River Biotechnology Co., Ltd., Beijing, China, and housed in an SPF‐grade animal facility with a 12‐h light/dark cycle. All animal experiments were approved by the Ethics Committee of the First Affiliated Hospital of Zhengzhou University (2025‐KY1342‐002) and strictly followed the National Institutes of Health's “Guidelines for the Housing and Use of Laboratory Animals.” The CCA mouse model was constructed as previously described [53]. Specifically, 20 µg domain of the NOTCH1 receptor (NICD), 4 µg myr‐AKT‐HA, and 1 µg SB100X transposase were diluted in 2 mL of 0.9% NaCl, aseptically filtered, and injected into the lateral tail vein over 5–7 s.

One week after injection, mice were randomly divided into two groups (*n* = 7 in each group): (1) PBS group, which received 200 µL of PBS by gavage daily; (2) *B.p* treatment group, which received 200 µL of PBS by gavage daily, containing 1 × 10^8^ CFU of bacteria. Treatment lasted for 4 weeks. At the experimental endpoint (5 weeks post‐injection), mice were sacrificed, livers were removed, weighed, and photographed. Liver tissue was fixed with 4% paraformaldehyde for histological analysis or flash‐frozen in liquid nitrogen for molecular biological studies. Colon tissue was collected and immunofluorescence stained.

### 5.5 Statistical Analysis

The alpha diversity (Shannon index, Simpson index and Invsimpson index) in each sample was calculated to estimate the gut microbiota richness by R (v4.2.0, vegan package [54]), the difference in alpha diversity were estimated with MaAsLin2 [55]. MaAsLin2 was used with disease status as the main fixed effect, adjusting for age, sex, BMI and comorbidities; cohort/center was included to account for between‐cohort heterogeneity (when applicable). Multiple testing was controlled by FDR. ″To deduce the community diversity between samples, we calculate the beta diversity (Bray–Curtis distance). PCoA [56] was analyzed to show the differences in microbial community structure among samples using Bray–Curtis distance. Covariate‐aPCoA approach was used to evaluate the influence of random effects on the relationship between gut microbiota and CCA [57].

Microbial community batch effects among cohorts were corrected with a Combat‐like extended method using MMUPHin [58]. The associations between each microbiome features and CCA were calculated using linear discriminant analysis (LDA) effect size (LEfSe) [59] (LDA score >2, and *p*‐value< 0.05), Wilcoxon rank‐sum test (*p*‐value <0.05), and MaAsLin2 (*p*‐value < 0.05). Co‐abundance network analysis was conducted using Spearman correlations between species, which significantly different between CCA group and Control group and only the significant correlations (*p*‐value < 0.05) larger than 0.4 or smaller than −0.4 were used for network construction. The networks were drawn by Cytoscape software. Significantly different clinical characteristics were calculated with Pearson's Chi‐square test or Wilcoxon rank sum test. Canonical Correspondence Analysis (R 4.2.0, vegan package [54]) was performed on the species profiling and redundancy analysis (R 4.2.0, vegan package [54]) was performed on the MetaCyc‐pathway profiling of the samples to assess the effect of each clinical characteristics. Spearman's correlation was used to describe the specific correlation between clinical indices and differential enriched features.

To identify the optimal machine learning algorithm for CCA diagnosis, we systematically evaluated 17 different algorithms using the same feature set and training/testing framework. The evaluated algorithms included: random forest (RF), Gradient Boosting Machine (GBM), Extreme Gradient Boosting (XGBoost), Recursive Partitioning and Regression Trees (rpart), Ridge Regression, Lasso Regression, Elastic Net (Enet) with varying alpha parameters (*α* = 0.1, 0.2, 0.3, 0.4, 0.5, 0.6, 0.7, 0.8, 0.9), Generalized Linear Model (glm), SVM. For each algorithm, we calculated AUC values with 95% confidence intervals for all datasets and computed the mean AUC across the three test sets to assess generalization performance. The algorithm with the highest average AUC across all test sets was defined as the optimal algorithm, and subsequent model building was based on this.

Based on significantly different species between CCA and Control group, we independently constructed predictive models using three distinct algorithms, including RF algorithms, WRF algorithms [60], and balanced random forest (BRF) algorithms [61]. For each model, first, fivefold cross‐validation was performed on the Hangzhou training set and species‐sets with a minimum error were chosen as the optimal set. The optimal species‐sets (including both CCA‐enriched and Control‐enriched species) were used to construct unconstrained models (model 1: model). Second, the probability of the test cohort (including Hangzhou test set, Zhengzhou test set, Xinjiang test set) was calculated using this set of species and an ROC was drawn by R (v4.2.0, pROC package).

Meanwhile, we conducted the same analysis based on the significantly different species that were enriched in CCA group. The selecting features enriched in CCA were used to construct enrichment‐constrained models (model 2: model constrained to CCA‐enriched features) [23]. This analysis was also performed based on the MetaCyc‐pathway level.

### 5.6 External Validation of the Metagenomic Classifiers

To further evaluate the disease specificity of the trained models, we obtained predictions for samples from other available gut metagenomic datasets (Table S1), as in previous studies [23, 25]. We screened publicly available fecal metagenomic datasets from published case‐control or cohort studies, resulting in data from 31 studies covering 6847 samples in 11 disease states for subsequent studies. We downloaded raw sequencing data from the European Nucleotide Archive and performed taxonomic analysis as described above. We selected the same feature set and used the same normalization parameters for data annotation, and then evaluated each external dataset using the trained CCA metagenomic classifier. FPR was used as the evaluation metric.

## Author Contributions

Z.G.R., S.S.Z., and Z.J.Y. designed the study. B.C.R., J.W.J., R.Q.Z., L.Z., W.Z.G., P.H.W., J.F.X., J.P., and T.A. collected clinical samples. H.F.L. and H.Z. extracted the bacterial DNA. D.Y.Z., C.C.Z., A.L., Z.L., and X.Z.J. analyzed the data. B.C.R. wrote the manuscript. All authors reviewed and approved the manuscript. The authorship order among co–first authors was assigned based on workload and manuscript preparation.

## Conflicts of Interest

The authors declare no conflicts of interest.

## Supporting information




**Supporting File 1**: advs74742‐sup‐0001‐SuppMat.docx

**Supporting File 2**: advs74742‐sup‐0002‐TableS1‐S34.xlsx

## Data Availability

The raw Illumina read data for all samples were available through the European Nucleotide Archive (ENA) at the European Bioinformatics Institute (EBI) under accession number PRJNA1111706. The clinical data of this study are available within the paper and its Supplementary Material.
