# Supplementary material for: Multicohort Validation of Gut Microbiome Signatures for Cholangiocarcinoma Diagnosis and Functional Characterization of Bifidobacterium Pseudocatenulatum
Source: Adv Sci (Weinh). 2026 Mar 12;13(27):e17658. doi: 10.1002/advs.202517658 (PMC13170203; doi:10.1002/advs.202517658)
Supplement: Supplementary file 1 — Supporting File 1: advs74742‐sup‐0001‐SuppMat.docx [file ADVS-13-e17658-s002.docx]

Supplementary material

Multicohort validation of gut microbiome signatures for cholangiocarcinoma diagnosis and functional characterization of *Bifidobacterium pseudocatenulatum*

Benchen Rao^1,2†^, Jianwen Jiang^3†^, Ruiqing Zhang^4†^, Dongya Zhang^5†^, Chenchen Zhang^5†^, Ang li^1,2^, Haifeng Lu^6^, Hua Zhang^6^, Lin Zhou^3^, Wenzhi Guo^7^, Peihao Wen^7^, Jianfeng Xue^7^, Jie Pan^7^, Tuerganaili Aji^4^, Zhou Lan^5^, Xianzhi Jiang^5^, Shusen Zheng^3^, Zujiang Yu^1,2^, Zhigang Ren^1,2*^

Supplementary material

1. Consent informed
2. **Supplementary Methods**
3. Supplementary Figure legend and Supplementary Table legend

Consent informed

**Informed consent form for scientific research**

**(Translated from Chinese)**

Dear participants,

We are from Department of Infectious Diseases, the First Affiliated Hospital of Zhengzhou University. We will free of charge help you monitor your healthy condition and record your clinical information and healthy/disease status or disease progression process. The collected tongue coating samples from participants in hospital will be used for scientific research. These results and data from the hospital electronic medical records will provide auxiliary data for clinical diagnosis and treatment, and will be used for scientific research. Thank you for your corporation.

Number: Diagnosis:

The information that we collect from this research project will be kept confidential. Information about you that will be collected during the research will be put away and no-one but the researchers will be able to see it. Any information about you will have a number on it instead of your name. Only the researchers will know what your number is and we will lock that information up with a lock and key. It will not be shared with or given to anyone except our research team.

The knowledge that we get from doing this research will be shared with you through community meetings before it is made widely available to the public. Confidential information will not be shared. There will be small meetings in the community and these will be announced. After these meetings, we will publish the results in order that other interested people may learn from our research.

I have read the foregoing information, or it has been read to me. I have had the opportunity to ask questions about it and any questions that I have asked have been answered to my satisfaction. I consent voluntarily to participate as a participant in this research.

Print Name of Participant__________________

Signature of Participant ___________________

Date ___________________________

Day/month/year

A literate witness must sign (if possible, this person should be selected by the participant and should have no connection to the research team). Participants who are illiterate should include their thumb-print as well.

I have witnessed the accurate reading of the consent form to the potential participant, and the individual has had the opportunity to ask questions. I confirm that the individual has given consent freely.

Print name of witness_____________________ AND Thumb print of participant

Signature of witness ______________________

Date ________________________

Day/month/year

Statement by the researcher/person taking consent

I have accurately read out the information sheet to the potential participant, and to the best of my ability made sure that the participant understands that the following will be done:

1. We will free of charge help you monitor your healthy condition and record your clinical information and healthy/disease status or disease progression process.

2. These data from hospital electronic medical records will be used for scientific research.

3. The collected tongue coating, fecal, and serum samples will be used for scientific research.

I confirm that the participant was given an opportunity to ask questions about the study, and all the questions asked by the participant have been answered correctly and to the best of my ability. I confirm that the individual has not been coerced into giving consent, and the consent has been given freely and voluntarily.

 A copy of this ICF has been provided to the participant.

Print Name of Researcher/person taking the consent________________________

Signature of Researcher /person taking the consent__________________________

Date ___________________________

Day/month/year

**Supplementary Methods**

**Inclusion and exclusion criteria of participants**

Patients with CCA complications such as biliary infection and biliary hemorrhage were excluded. All participants who had the following diseases were excluded: Other primary tumors, other tumors that have metastasized to the liver or bile duct. In addition, all participants who received antibiotics and/or probiotics within 8 weeks before providing samples were also excluded.

**Healthy controls recruitment and screening criteria**

Healthy controls were recruited from the Health Management Centers of the respective hospitals. All potential participants underwent comprehensive health screening including: a) Physical examination and medical history review to exclude individuals with any current or previous malignancies, liver diseases (including viral hepatitis, cirrhosis, fatty liver disease, autoimmune liver diseases), biliary tract diseases, gastrointestinal disorders (inflammatory bowel disease, irritable bowel syndrome, chronic gastritis), or metabolic diseases (diabetes mellitus, obesity with BMI ≥ 28 kg/m²); b) Laboratory tests including liver function (ALT, AST, total bilirubin, albumin), renal function (creatinine, urea nitrogen), blood glucose, and complete blood count to confirm normal physiological status; c) Abdominal ultrasonography to exclude hepatobiliary abnormalities; d) Exclusion of individuals who had received antibiotics, probiotics, prebiotics, or any medications known to affect gut microbiota within 8 weeks prior to fecal sample collection; e) Exclusion of individuals with recent acute infections, chronic inflammatory diseases, or immunocompromised states. All healthy controls provided written informed consent.

**Sample collection**

The fecal samples were provided from each participant between 6:30 am and 8:30 am. The fecal samples were stored in low-temperature cryogenic vials and quickly transferred to the -80°C freezer. All samples that were at room temperature for more than 2 hours were excluded.

**DNA extraction and Library construction**

The microbial DNA in fecal samples were extracted by the Qiagen Mini Kit (Qiagen, Hilden, Germany) as described previously[1]. The samples were processed by phenol trichloromethane DNA extraction using a bead beater to mechanically disrupt cells, followed by phenol–chloroform extraction. Then, the DNA was purified according to the manufacturer's instructions. The DNAs were quantified by the Qubit 2.0 Fluorometer (Invitrogen, Carlsbad, CA, USA), and molecular size was estimated using agarose gel electrophoresis. All microbial DNAs were diluted to 10 ng/μL for microbial analysis. DNA library construction was performed based on the standard protocols, as our previous study[2].

**Bacterial culture**

*Bifidobacterium pseudocatenulatum* (BNCC134343), *Bifidobacterium adolescentis* (BNCC185974), *Bifidobacterium longum* (BNCC341605) and Bifidobacterium broth (BBL) (BNCC352254) were obtained from BeNa Culture Collection (BNCC). Bacteria were cultured anaerobically in BBL medium at 37°C for 48 hours in an anaerobic chamber containing 5% H2, 5% CO2, and 90% N2. Bacterial concentrations were determined by measuring optical density at 600 nm and confirmed by colony-forming unit (CFU) counting on Bifidobacterium agar.

**Cell culture**

Human cholangiocarcinoma cell lines HCCC-9810 and RBE were obtained from the Cell Bank of the Chinese Academy of Sciences (Shanghai, China) and authenticated by short tandem repeat profiling. HCCC-9810 and RBE cells were cultured in RPMI-1640 medium (Gibco, Grand Island, NY, USA) supplemented with 10% fetal bovine serum (FBS, Gibco) and 1% penicillin-streptomycin (Gibco). All cells were maintained at 37°C in a humidified atmosphere containing 5% CO₂ and routinely tested for mycoplasma contamination.

**Cell viability assay**

Cell viability was conducted using the Cell Counting Kit-8 (CCK-8, Dojindo Molecular Technologies, Kumamoto, Japan) according to the manufacturer's protocol. Briefly, cells were seeded in 96-well plates at a density of 3,000 cells per well and cultured overnight. Cells were then treated with different concentrations (0%, 0.05%, 0.1%, 0.5%, 1%, 5%, 10%, 20%) of bacterial CM for 48 hours. Subsequently, 10% of CCK-8 solution was added to each well and incubated for 2 hours at 37°C. Absorbance was measured at 450 nm using a microplate reader.

**Colony formation assay**

Cells were seeded in 6-well plates at a density of 1,000 cells per well and treated with 10% B.P CM or BBL for 10-14 days, with medium refreshed every 3 days. Colonies were fixed with 4% paraformaldehyde (Sigma-Aldrich) for 15 minutes and stained with 0.1% crystal violet (Sigma-Aldrich) for 30 minutes at room temperature. After washing with PBS, colonies containing >50 cells were counted under a light microscope.

**Wound healing assay**

Cells were seeded in 6-well plates and cultured to 90–95% confluence. A straight line was drawn on the cell monolayer by a sterile 200 μl pipette tip. Cells were washed twice with PBS to remove detached cells and then cultured in serum-free medium containing 10% bacterial CM or BBL. Images were acquired at 0 and 48 hours by an inverted microscope (Olympus, Tokyo, Japan). Wound healing rate was calculated using ImageJ software (National Institutes of Health, Bethesda, MD) according to the formula: Migration rate = [(Initial wound area - Remaining wound area) / Initial wound area] × 100%.

**Transwell invasion assay**

Cell invasion ability was assessed using 24-well Transwell chambers with 8 μm pore size polycarbonate membranes (Corning, NY, USA). 50 μl of Matrigel (1:8 dilution, BD Biosciences) was coated onto the upper surface of the membrane and incubated at 37°C for 30 min. Cells (8*10^4) in serum-free medium containing 10% bacterial CM or BBL were added to the upper chamber, 600 μl of complete medium containing 20% ​​fetal bovine serum was added to the lower chamber as a chemokine. After 48 hours of incubation, uninvaded cells on the upper surface were removed with a swab. Invaded cells on the lower surface were fixed with 4% paraformaldehyde for 15 min and stained with 0.1% crystal violet for 30 min. Photographs were taken under an optical microscope, and the number of invading cells was counted using ImageJ software.

**TUNEL staining**

According to the instructions, apoptotic cells were detected using the TUNEL Apoptosis Assay Kit (MeilunBio, China). In short, cells were treated with bacterial CM, BBL, PBS, or acetate for 48 hours. Cells were fixed with 4% paraformaldehyde for 30 minutes, permeabilized with 0.3% Triton X-100 in PBS for 5 minutes, and then incubated with the TUNEL reaction mixture at 37°C in the dark for 1 hour. After mounting with anti-fluorescence attenuation mounting medium, observe under a fluorescence microscope. The percentage of TUNEL-positive cells was calculated.

**Immunohistochemistry**

Paraffin-embedded tissue sections were dewaxed with xylene and rehydrated with graded ethanol. Antigen retrieval was performed by boiling in 10 mM citrate buffer (pH 6.0) for 20 minutes. Endogenous peroxidase activity was measured by blocking with 3% H2O2 for 10 minutes. After blocking with 5% bovine serum albumin (BSA, Sigma-Aldrich) at room temperature for 1 hour, sections were incubated overnight at 4°C with anti-Ki67 antibody (1:1000, ab16667, GB151499, Servicebio). Subsequently, sections were incubated with horseradish peroxidase (HRP)-labeled secondary antibody (1:200, GB23303, Servicebio) at room temperature for 1 hour. Staining was performed using 3,3'-diaminobenzidine (DAB, Dako, Glostrup, Denmark) and counterstained with hematoxylin. Images were acquired using an optical microscope (Olympus), and Ki67-positive cells were quantified for each high-power field using ImageJ software.

**Immunofluorescence staining**

Paraffin-embedded colon tissue sections were dewaxed, rehydrated, and subjected to antigen retrieval as described above. After blocking with 5% BSA for 1 hour, the sections were incubated overnight at 4°C with the following primary antibodies: anti-Claudin 1 antibody (1:500, GB152543, Servicebio), anti-Occludin antibody (1:300, GB111401, Servicebio), or anti-ZO-1 antibody (1:200, GB15195, Servicebio). Subsequently, the sections were incubated at room temperature in the dark for 1 hour with CY3-labeled secondary antibody (1:300, GB21303, Servicebio). Finally, the cell nuclei were counterstained with DAPI for 5 minutes. Fluorescence images were acquired using a confocal microscope (Obercohen Zeiss LSM 880, Germany), and the average fluorescence intensity of the slices was quantified using ImageJ software.

**Western blot analysis**

Total protein was extracted from tumor tissues using RIPA lysis buffer (Solarbio, China) supplemented with a mixture of protease inhibitors (MCE, China) and a mixture of phosphatase inhibitors (MCE, China).

Protein were separated by 8–12% sodium dodecyl sulfate-polyacrylamide gel electrophoresis (SDS-PAGE) and then transferred to PVDF membrane (Millipore, USA). After blocking, the membranes were incubated with specific primary and corresponding secondary antibodies (see Supplementary Table 34 for details of the primary antibodies used in Western blotting experiments), and finally visualized by Odyssey Infrared Imaging System (LI-COR Bioscience, NE) or LumiGLO enhanced chemiluminescent (ECL). Band intensity was quantified using ImageJ software.

**Reference**

[1] G. Y. Cui, B. C. Rao, Z. H. Zeng, X. M. Wang, T. Ren, H. Y. Wang, H. Luo, H. Y. Ren, C. Liu, S. Y. Ding, J. J. Tan, Z. G. Liu, Y. W. Zou, Z. G. Ren, Z. J. Yu, *Mil Med Res* **2022**, *9* (1), 32, <https://doi.org/10.1186/s40779-022-00387-y>.

[2] Z. Ren, A. Li, J. Jiang, L. Zhou, Z. Yu, H. Lu, H. Xie, X. Chen, L. Shao, R. Zhang, S. Xu, H. Zhang, G. Cui, X. Chen, R. Sun, H. Wen, J. P. Lerut, Q. Kan, L. Li, S. Zheng, *Gut* **2018**, <https://doi.org/10.1136/gutjnl-2017-315084>.

**Supplementary figure legend**





**Figure S1.** **Gut microbial diversity and composition based on species level in CCA and Control subjects.** (A) Shannon index, (B) Simpson index and (C) Invsimpson index were calculated for α-diversity at the species level (*p*-values from Wilcoxon rank-sum tests and MaAsLin2 were shown). β-diversity was displayed by (D) Bray-Curtis distance and (E) anosim on Bray-Curtis distance. (F) PCoA and (G) aPCoA of species level based on Bray-Curtis distance. (H) Relative abundance of the top 20 most abundant species of the samples in CCA and Control groups. MaAsLin2, Microbiome Multivariable Association with Linear Models; PCoA, Principal co-ordinates analysis; aPCoA, Covariate-adjusted principal coordinate analysis; CCA, cholangiocarcinoma.





**Figure S2. Gut microbial diversity and composition based on genus level in CCA and Control subjects.** (A) Shannon index, (B) Simpson index and (C) Invsimpson index were calculated for α-diversity at the genus level (*p*-values from Wilcoxon rank-sum tests and MaAsLin2 were shown). (D) PCoA and (E) aPCoA of genus level based on Bray-Curtis distance. (F) Relative abundance of the top 20 most abundant genera of the samples in CCA and Control groups. PCoA, Principal co-ordinates analysis; aPCoA, Covariate-adjusted principal coordinate analysis; CCA, cholangiocarcinoma.





**Figure S3. Significantly different gut bacteria between CCA and Control subjects at genus level.** (A) Detection frequency and LDA score of the significantly different gut bacteria (genus level) in CCA and Control groups using Wilcoxon rank sum test, LEfSe and MaAsLin2 were shown. (B) Co-occurrence network was deduced from significantly different genera. Nodes depict species with their taxonomic information displayed in the center and colored according to phylum. The size of the nodes indicated relative abundance of the genera. Connecting lines represent Spearman correlation coefficient values >=0.4 (green) or <= -0.4 (purple). LDA, linear discriminant analysis; LEfSe, linear discriminant analysis effect size; MaAsLin2, Microbiome Multivariable Association with Linear Models; CCA, cholangiocarcinoma.


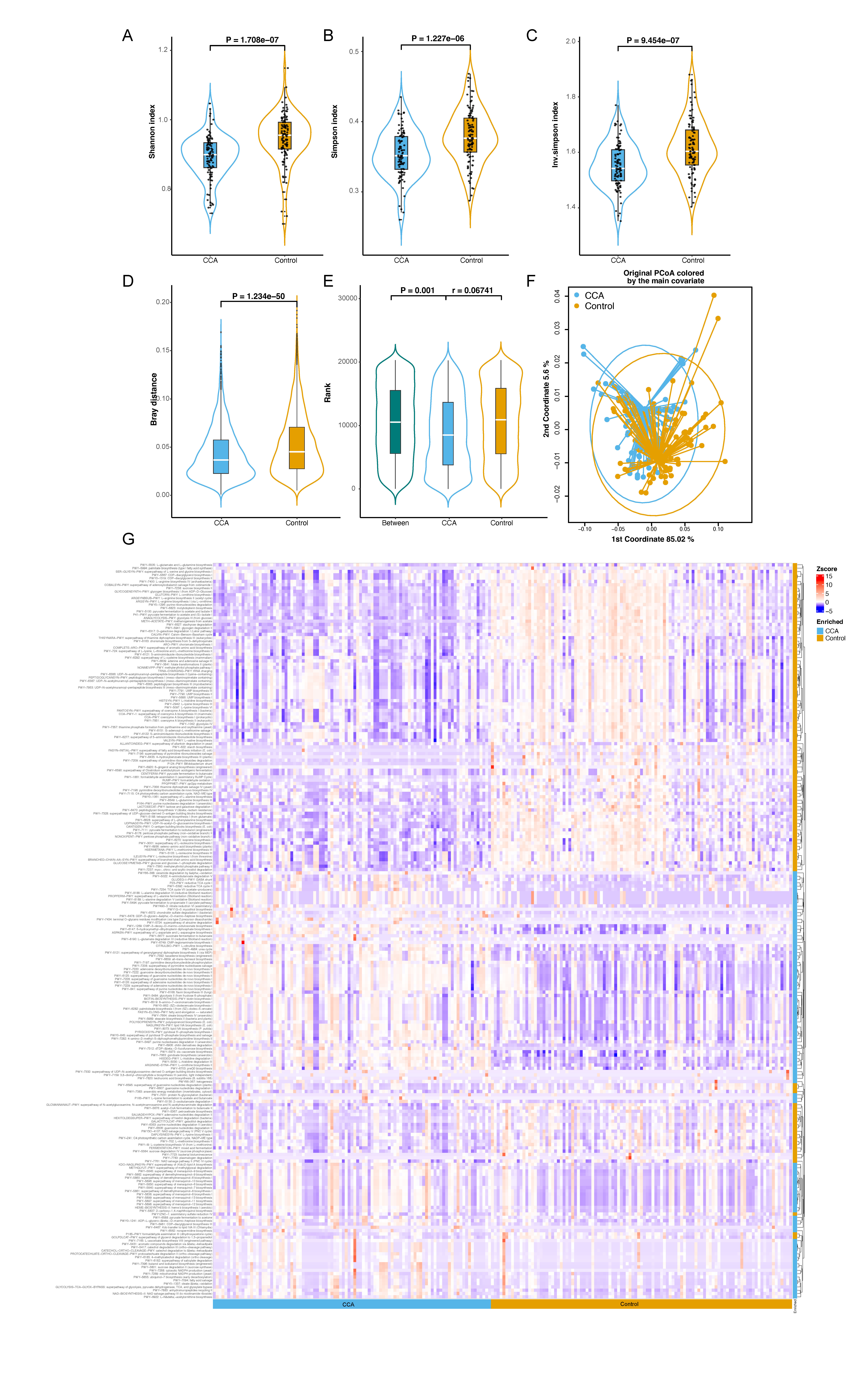


**Figure S4. Significantly different MetaCyc-pathways between CCA and Control subjects.** (A) Shannon index, (B) Simpson index and (C) Invsimpson index were calculated for α-diversity at the MetaCyc-pathways level (*p*-values from Wilcoxon rank-sum tests and MaAsLin2 were shown). β-diversity was displayed by (D) Bray-Curtis distance and (E) anosim on Bray-Curtis distance. (F) PCoA of MetaCyc-pathways level based on Bray-Curtis distance. (G) Heatmap of the Relative abundances of the significantly different MetaCyc-pathways using Wilcoxon rank-sum test (*p-*value < 0.01) and MaAsLin2 was shown. MaAsLin2, Microbiome Multivariable Association with Linear Models; PCoA, Principal co-ordinates analysis; aPCoA, Covariate-adjusted principal coordinate analysis; CCA, cholangiocarcinoma.





**Figure S5. Systematic comparison of 17 machine learning algorithms for CCA diagnosis.** ROC curves showing the performance of 16 machine learning algorithms (in addition to Random Forest presented in figure 5) across training and test sets. Algorithms evaluated include: Random Forest (RF), Gradient Boosting Machine (GBM), Extreme Gradient Boosting (XGBoost), Recursive Partitioning and Regression Trees (rpart), Ridge Regression, Lasso Regression, Elastic Net (Enet) with varying alpha parameters (α = 0.1, 0.2, 0.3, 0.4, 0.5, 0.6, 0.7, 0.8, 0.9), Generalized Linear Model (glm), Support Vector Machine (SVM).





**Figure S6. The diagnostic performance of the best model-1 and model-2.** “WRF-species-model-1” was selected as the best model-1 for CCA. (A) Twenty-two species markers were selected to establish “WRF-species-model-1”. (B) The mean decrease in accuracy for “WRF-species-model-1”. Probability of diseases between CCA and Control in (C) training phase from Hangzhou, (D) testing phase from Hangzhou, (E) external testing phase from Zhengzhou, (F) external testing phase from Xinjiang. “RF-species-model-2” was selected as the best enrichment-constrained models (model-2) for CCA. (G) Eight species markers were selected to establish “RF-species-model-2”. (H) The mean decrease in accuracy for “RF-species-model-2”. Probability of diseases between CCA and Control in (I) training phase from Hangzhou, (J) testing phase from Hangzhou, (K) external testing phase from Zhengzhou, (L) external testing phase from Xinjiang. Model 1, unconstrained models; Model 2, enrichment-constrained models based on CCA-enriched features; WRF-species-model-1, unconstrained model based on significantly different species by weighted random forest algorithms; RF-species-model-2, enrichment-constrained models based on CCA-enriched species by random forest algorithms; POD, probability of diseases (CCA); CCA, cholangiocarcinoma.





**Figure S7. Gut microbial diagnostic** **enrichment-constrained models (model-2) for CCA.** The optimal CCA-enriched species-sets were used to enrichment-constrained models by (A) RF algorithms, (B) WRF algorithms and (C) BRF algorithms. The optimal CCA-enriched pathway-sets were used to enrichment-constrained models by (D) RF algorithms, (E) WRF algorithms and (F) BRF algorithms. The “RF-species-model-2” were further evaluated to distinguish between CCA and HCC. (G) ROC curve, (H) prediction performance and (I) POD index of “RF-species-model-2” to distinguish between CCA and HCC. Model 2, enrichment-constrained models based on CCA-enriched features; RF algorithms, random forest algorithms; WRF algorithms, weighted random forest algorithms; BRF algorithms, balanced random forest algorithms; POD, probability of diseases (CCA); RF-species-model-2, enrichment-constrained models based on CCA-enriched species by random forest algorithms; HCC, hepatocellular carcinoma; CCA, cholangiocarcinoma.


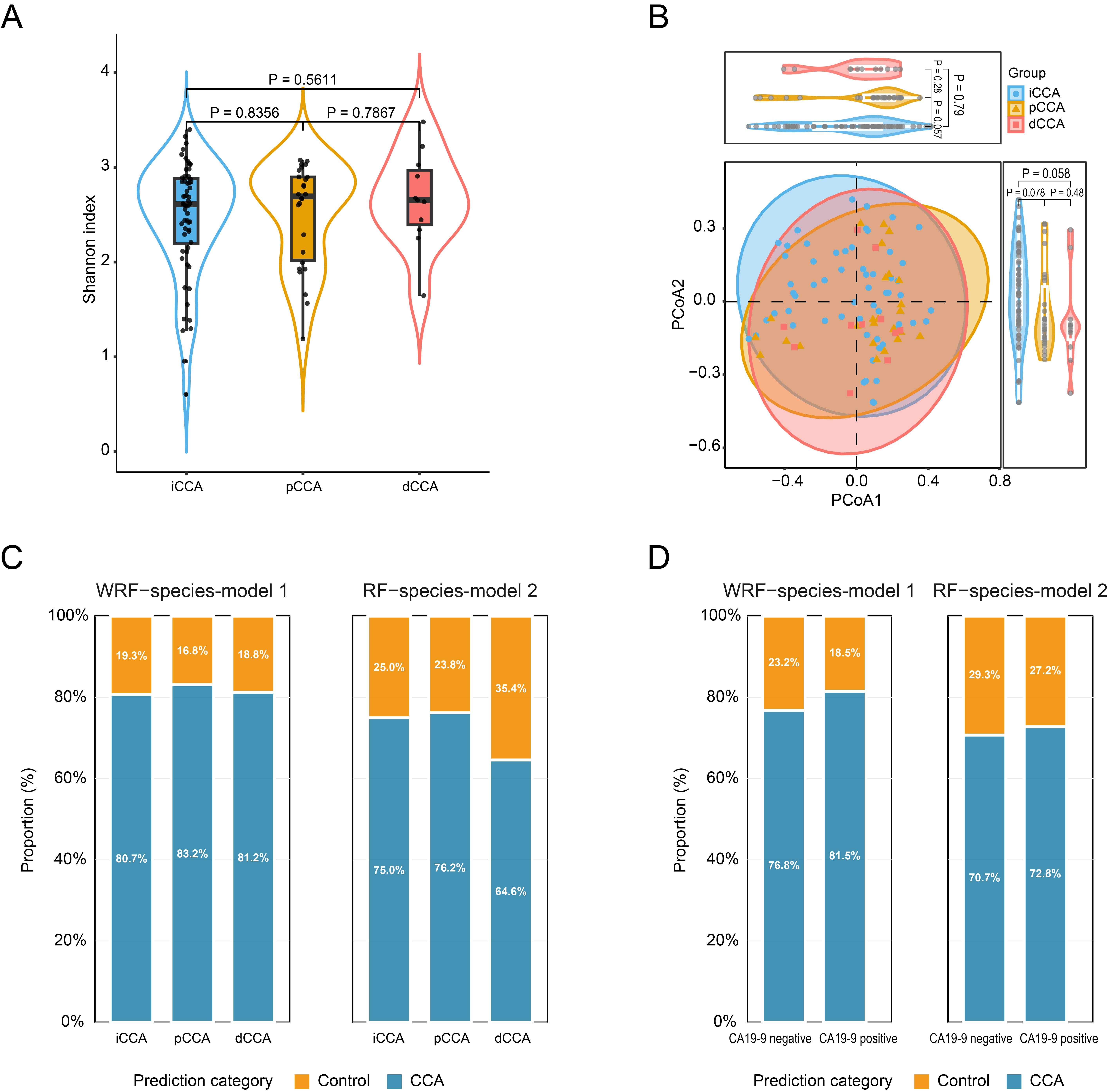


**Figure S8. Diagnostic performance across CCA subtypes and CA19-9 status.** (A) Alpha diversity analysis and (B) PCoA among iCCA, (n=140), pCCA (n=101), and dCCA (n=48)**.** (C) Performance of WRF-species-model-1 and RF-species-model-2 (right) across anatomical CCA subtypes. (D) Performance of WRF-species-model-1 and RF-species-model-2 in CA19-9-negative (<37 U/mL, n=82) and CA19-9-positive (≥37 U/mL, n=184) CCA patients. Blue bars indicate correct CCA classification; orange bars indicate misclassification as controls.





**Figure S9. “WRF-species-model-1” performance in discriminating CCA from benign hepatobiliary diseases.** (A) Validation using liver fibrosis cohort. (B) Validation using NAFLD cohort from USA (PRJNA373901). (C) Validation using NAFLD cohort from Italy (PRJEB14215). (D) Validation using NAFLD cohort from Sweden (PRJNA420817). WRF-species-model-1, unconstrained model based on significantly different species by weighted random forest algorithms; RF-species-model-2, enrichment-constrained models based on CCA-enriched species by random forest algorithms; POD, probability of diseases (CCA); CCA, cholangiocarcinoma.





**Figure S10. “RF-species-model-2” performance in discriminating CCA from benign hepatobiliary diseases.** (A) Validation using liver fibrosis cohort. (B) Validation using NAFLD cohort from USA (PRJNA373901). (C) Validation using NAFLD cohort from Italy (PRJEB14215). (D) Validation using NAFLD cohort from Sweden (PRJNA420817). WRF-species-model-1, unconstrained model based on significantly different species by weighted random forest algorithms; RF-species-model-2, enrichment-constrained models based on CCA-enriched species by random forest algorithms; POD, probability of diseases (CCA); CCA, cholangiocarcinoma.


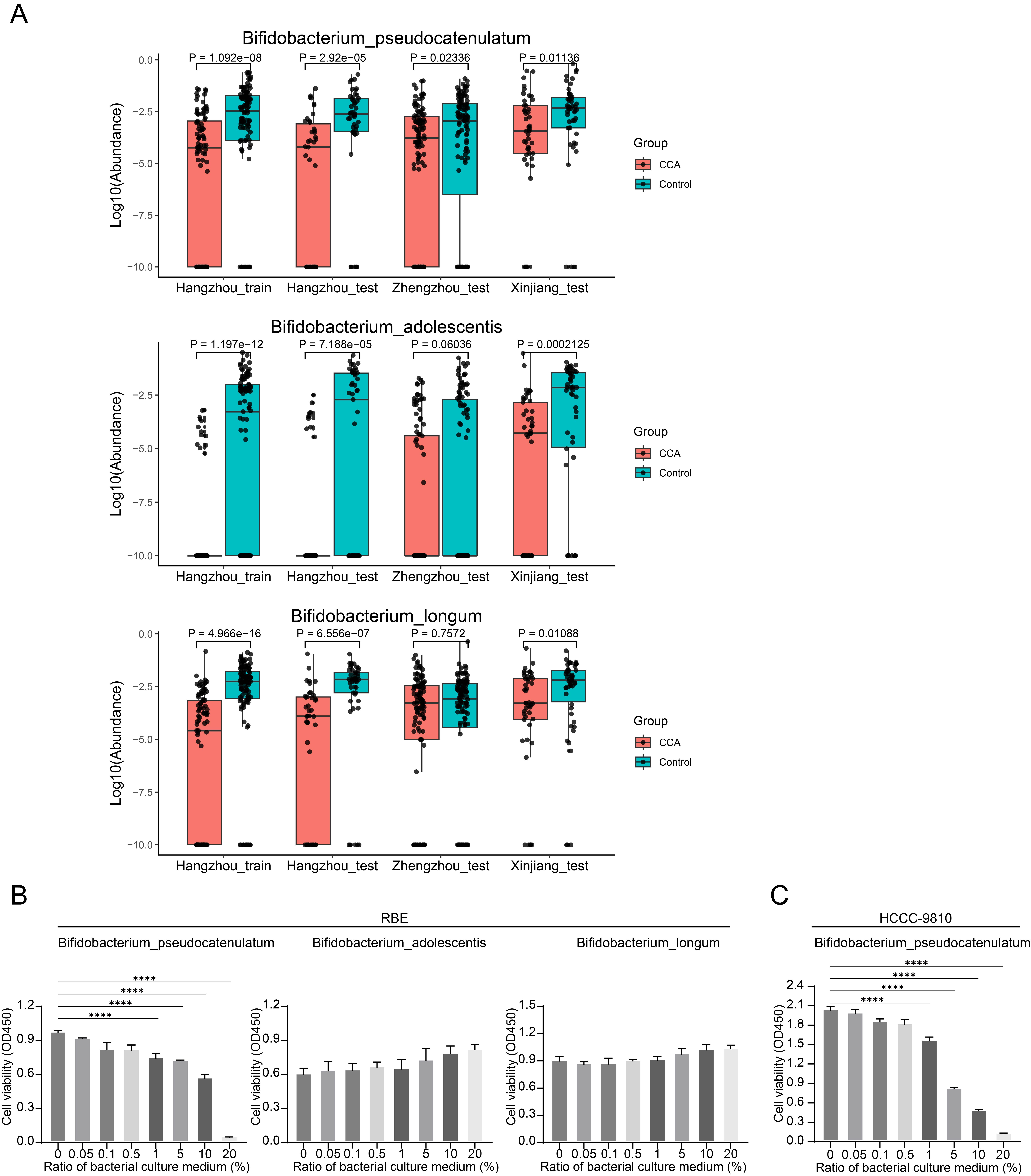


**Figure S11: *Bifidobacterium pseudocatenulatum* exhibits anti-CCA activity in vitro.** (A) Relative abundance of *B. pseudocatenulatum*, *B. adolescentis*, and *B. longum* across four cohorts (Wilcoxon rank-sum test). (B) Dose-dependent effects of conditioned media from three Bifidobacterium species on RBE cell viability. (C) Dose-dependent effects of conditioned media from *B. pseudocatenulatum* on HCCC-9810 cell viability. CCA, cholangiocarcinoma.


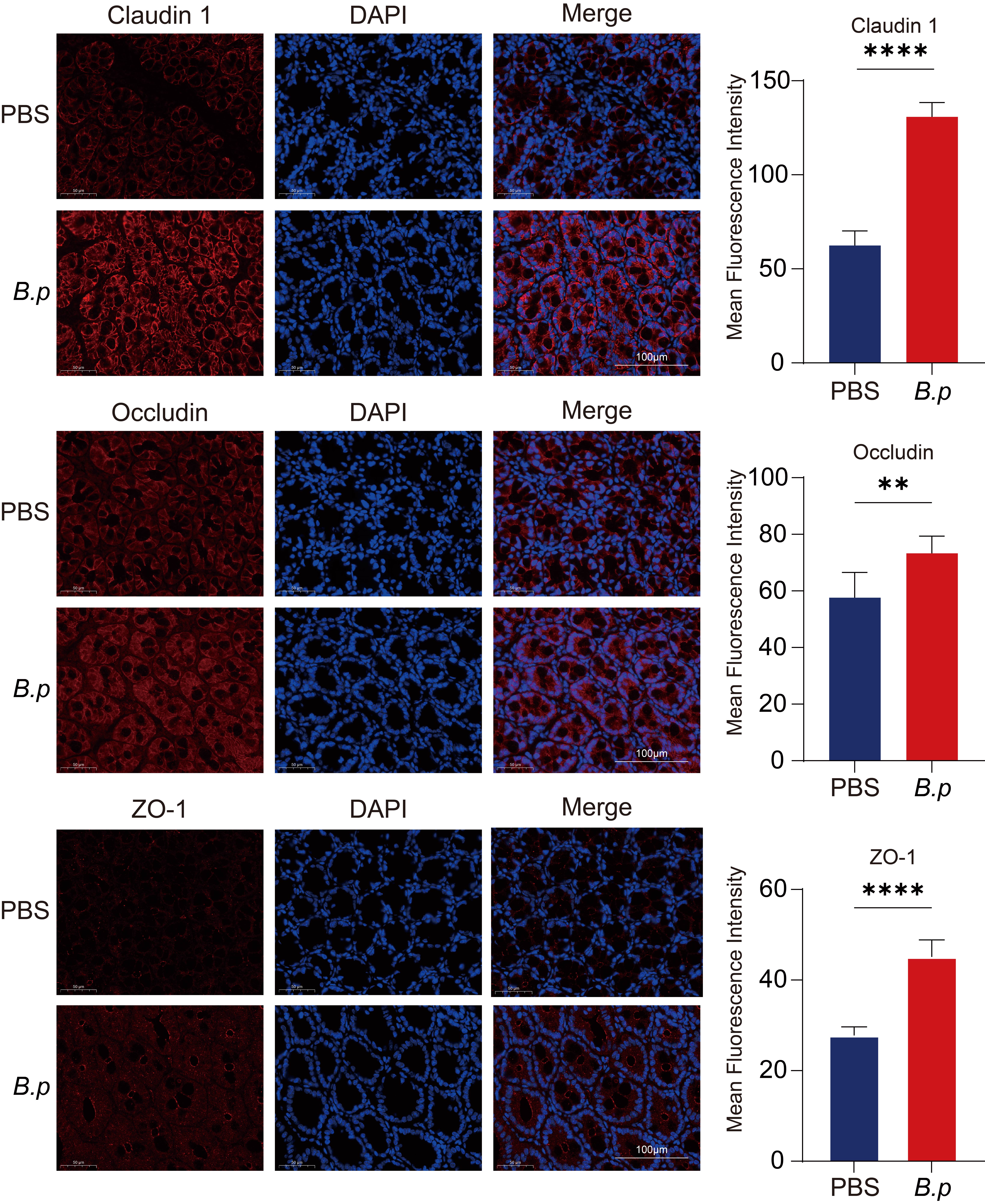


**Figure S12: *B. pseudocatenulatum* enhances intestinal barrier integrity in CCA mice.** Immunofluorescence staining and quantification of tight junction proteins Claudin 1, Occludin, and ZO-1 in colonic tissues from PBS- and *B.p*-treated mice.

**Supplementary Table legend**

**Table S1.** Detailed information of publicly datasets used for external validation of the metagenomic classifiers. CRC, colorectal cancer; T2D, type 2 diabetes; T1D, type 1 diabetes; CD, Crohn’s disease; UC, ulcerative colitis; LD, liver diseases; ACD, atherosclerotic coronary disease; PC, pancreatic cancer; BRCA, breast cancer; CP, chronic pancreatitis; CTR, healthy controls; CCA, cholangiocarcinoma.

**Table S2.** Raw data of the clinical characteristics of participants. DB, direct bilirubin; TB, total bilirubin; GLO, globulin; ALB, albumin; IB, indirect bilirubin; TP, total protein; PLT, platelets; ALT, Alanine aminotransferase; AST, Aspartate aminotransferase; CTP, Child-Turcotte-Pugh; GGT, Glutamyl transpeptidase; CA19-9, Carbohydrate antigen 19-9; CHB, chronic hepatitis B; T2DM, type 2 diabetes; CAD, Coronary heart disease; LC, liver cirrhosis; CCA, cholangiocarcinoma.

**Table S3.** Comparison of the gut microbial community between CCA and Control group at species level. LDA, linear discriminant analysis; CCA, cholangiocarcinoma.

**Table S4.** Comparison of the gut microbial community between CCA and Control group at genus level. LDA, linear discriminant analysis; CCA, cholangiocarcinoma.

**Table S5.** Comparison of the gut microbial community between CCA and Control group at metacyc-pathway level. LDA, linear discriminant analysis; CCA, cholangiocarcinoma.

**Table S6.** Canonical correspondence analysis on species. CCA, cholangiocarcinoma.

**Table S7.** Redundancy analysis on metacyc-pathways. CCA, cholangiocarcinoma.

**Table S8.** R values of the spearman's rank correlation analysis between gut microbiome (species, genus and metacyc pathways) and clinical phenotypes.

**Table S9.** P values of the spearman's rank correlation analysis between gut microbiome (species, genus and metacyc pathways) and clinical phenotypes.

**Table S10.** Twenty-two Species used to construct the RF-species-model-1.

**Table S11.** Predict probability in the training phase from Hangzhou of RF-species-model-1. RF-species-model-1, unconstrained model based on significantly different species by random forest algorithms.

**Table S12.** Predict probability in the testing phase from Hangzhou of RF-species-model-1. RF-species-model-1, unconstrained model based on significantly different species by random forest algorithms.

**Table S13.** Predict probability in the test set from Zhengzhou of RF-species-model-1. RF-species-model-1, unconstrained model based on significantly different species by random forest algorithms.

**Table S14.** Predict probability in the test set from Xinjiang of RF-species-model-1. RF-species-model-1, unconstrained model based on significantly different species by random forest algorithms.

**Table S15.** Predict probability of WRF-species-model-1. WRF-species-model-1, unconstrained model based on significantly different species by weighted random forest algorithms.

**Table S16.** Predict probability of BRF-species-model-1. BRF-species-model-1, unconstrained model based on significantly different species by balanced random forest algorithms.

**Table S17.** Eighteen pathway markers used to construct the RF-pathway-model-1. RF-pathway-model-1, unconstrained model based on significantly different pathways by random forest algorithms.

**Table S18.** Predict probability of RF-pathway-model-1. RF-pathway-model-1, unconstrained model based on significantly different pathways by random forest algorithms.

**Table S19.** Predict probability of WRF-pathway-model-1. WRF-pathway-model-1, unconstrained model based on significantly different pathways by weighted random forest algorithms.

**Table S20.** Predict probability of BRF-pathway-model-1. BRF-pathway-model-1, unconstrained model based on significantly different pathways by balanced random forest algorithms.

**Table S21.** Eight Species used to construct the RF-species-model-2. RF-species-model-2, enrichment-constrained models based on CCA-enriched species by random forest algorithms.

**Table S22.** Predict probability of RF-species-model-2. RF-species-model-2, enrichment-constrained models based on CCA-enriched species by random forest algorithms.

**Table S23.** Predict probability of WRF-species-model-2. WRF-species-model-2, enrichment-constrained models based on CCA-enriched species by weighted random forest algorithms.

**Table S24.** Predict probability of BRF-species-model-2. BRF-species-model-2, enrichment-constrained models based on CCA-enriched species by balanced random forest algorithms.

**Table S25.** Predict probability of RF-pathway-model-2. RF-pathway-model-2, enrichment-constrained models based on CCA-enriched pathways by random forest algorithms.

**Table S26.** Predict probability of WRF-pathway-model-2. WRF-pathway-model-2, enrichment-constrained models based on CCA-enriched pathways by weighted random forest algorithms.

**Table S27.** Predict probability of BRF-pathway-model-2. BRF-pathway-model-2, enrichment-constrained models based on CCA-enriched pathways by balanced random forest algorithms.

**Table S28.** Predict probability of WRF-species-model-1 to distinguish CCA from HCC. WRF-species-model-1, unconstrained model based on significantly different species by weighted random forest algorithms.

**Table S29.** Predict probability of RF-species-model-2 to distinguish CCA from HCC. RF-species-model-2, enrichment-constrained models based on CCA-enriched species by random forest algorithms.

**Table S30.** Predict probability of WRF-species-model-1 to distinguish CCA from LF. LF, liver fibrosis; WRF-species-model-1, unconstrained model based on significantly different species by weighted random forest algorithms.

**Table S31.** Predict probability of RF-species-model-2 to distinguish CCA from LF. LF, liver fibrosis; RF-species-model-2, enrichment-constrained models based on CCA-enriched species by random forest algorithms.

**Table S32.** FDR for each external validation cohort from publicly datasets of WRF-species-model-1. WRF-species-model-1, unconstrained model based on significantly different species by weighted random forest algorithms. CRC, colorectal cancer; T2D, type 2 diabetes; T1D, type 1 diabetes; CD, Crohn’s disease; UC, ulcerative colitis; LD, liver diseases; ACD, atherosclerotic coronary disease; PC, pancreatic cancer; BRCA, breast cancer; CP, chronic pancreatitis; CTR, healthy controls; CCA, cholangiocarcinoma.

**Table S33.** FDR for each external validation cohort from publicly datasets of RF-species-model-2. RF-species-model-2, enrichment-constrained models based on CCA-enriched species by random forest algorithms. CRC, colorectal cancer; T2D, type 2 diabetes; T1D, type 1 diabetes; CD, Crohn’s disease; UC, ulcerative colitis; LD, liver diseases; ACD, atherosclerotic coronary disease; PC, pancreatic cancer; BRCA, breast cancer; CP, chronic pancreatitis; CTR, healthy controls; CCA, cholangiocarcinoma.

**Table S34.** Details of the rabbit anti-mouse primary antibody product in Western blot analysis.
